# Monolithic and scalable capacitive electrodes leveraging parasitic capacitance for full-body pressure mapping

**DOI:** 10.1126/sciadv.aeh8618

**Published:** 2026-10-30

**Authors:** Donho Lee, Hyeonseok Han, Chankyu Han, Seungtaek Jang, Changjoon Lee, Chang-yeon Gu, Jiwon Moon, Jihyeon Ahn, Junseong Ahn, Ji-Hwan Ha, Seokjoo Cho, Byeongmin Kang, Yong Suk Oh, Jungrak Choi, Inkyu Park

**Affiliations:** ^1^ Department of Mechanical Engineering, Korea Advanced Institute of Science and Technology (KAIST); Daejeon, 34141, Republic of Korea.; ^2^ Department of Electro-Mechanical Systems Engineering, Korea University; Sejong, 30019, Republic of Korea.; ^3^ Department of Mechanical Engineering, Hanbat National University; Daejeon, 34158, Republic of Korea.; ^4^ Department of Mechanical Engineering, Changwon National University; Changwon, 51140, Republic of Korea.; ^5^ Electronics and Telecommunications Research Institute (ETRI); Daejeon, 34129, Republic of Korea.

## Abstract

Scaling capacitive pressure sensor arrays to large areas remains challenging due to electrode alignment errors, fabrication complexity, and limited dynamic range inherent in conventional parallel-plate architectures. Here, we present a scalable capacitive pressure sensor array based on a monolithic electrode architecture, where transmitter and receiver electrodes are co-integrated into a single substrate. A deformable dielectric and a floating ground (GND) layer are positioned above, eliminating alignment errors and reducing the layer-stacking process by 20%. The parasitic capacitance between the electrodes and GND layer is intentionally incorporated into the sensing mechanism, enabling stable pressure detection with effective electromagnetic interference shielding. The resulting 2911-cell array (1.7 m by 1.0 m) achieves 30 Hz full-body pressure mapping with high linearity (R^2^ = 0.994). Demonstrations in sleep posture classification, pressure injury prevention, and exercise monitoring validate the platform’s versatility for healthcare and human-machine interfaces.

## INTRODUCTION

Real-time measurement of pressure distribution using sensor arrays (i.e., pressure mapping) across diverse interfaces including prostheses (*1*, *2*), internal organs (*3*), skin interfaces (*4*–*9*), and mattresses (*10*) enables personalized healthcare applications for rehabilitation, treatment, and continuous support. In particular, for full-body pressure monitoring, the development of large-area pressure sensor arrays (i.e., pressure mattresses) has become increasingly important in addressing critical healthcare needs, such as posture monitoring during sleep (*11*), respiration and heart rate monitoring (*11*), prevention of pressure injuries (*12*, *13*), and gait analysis (*14*). Unlike single pressure sensors attached to locations of interest, a large-area pressure sensor array for full-body coverage provides a comprehensive assessment of body pressure distribution, allowing early detection of abnormal pressure points and improving patient care outcomes.

In particular, healthcare applications and exercise monitoring, such as sleep posture classification, the prevention of pressure and musculoskeletal injuries, as well as gait analysis, require sensors capable of accurately measuring pressure induced by both static (e.g., prolonged pressure) and dynamic (e.g., physical movement) loadings. Among various types of pressure sensors, such as capacitive (*11*, *13*, *15*–*18*), resistive (*19*), and triboelectric sensors (*10*, *20*), capacitive pressure sensors exhibit superior response characteristics for measuring both static and dynamic loadings and provide reliable data acquisition under varying environmental conditions of temperature and humidity. In addition, capacitive pressure sensors have shown significant advantages in terms of high sensitivity and low power consumption for healthcare and industrial applications that require both precision and scalability (*17*, *21*). However, conventional parallel-plate capacitive pressure sensors face several challenges, particularly when implemented as large-area arrays. These include (1) signal noise caused by the deformation of electrode layers and exposed wired interconnections, (2) complex multilayer stacking that hampers scalable fabrication, and (3) difficulties in TX–RX alignment that lead to cell-to-cell non-uniformity in baseline capacitance (i.e., sensor-to-sensor variation, baseline capacitance variation). In addition, pressure-induced increases in TX–RX capacitance monotonically consume the available dynamic range, making array-level readout vulnerable to saturation and limiting stable large-area pressure mapping.

In this work, we present a large-area capacitive pressure sensor array featuring a structural architecture of capacitive electrodes (i.e., TX and RX) and leveraging parasitic capacitance, designed to enhance signal stability and manufacturing efficiency. The sensor array integrates both TX and RX electrodes into a monolithic, fixed electrode layer, over which a deformable dielectric layer and a rationally designed ground (GND) layer are sequentially stacked. The mattress-type sensor array measures full-body pressure distribution over a 1.7 m by 1.0 m area using 2911 sensing cells at a sampling rate of 30 Hz. Since the capacitive electrodes, consisting of TX and RX, are embedded and fixed in a single electrode layer, signal noise caused by the movement of deformable electrode layers and wired interconnections can be effectively suppressed, in contrast to conventional parallel-plate capacitive pressure sensors. In addition, the monolithic capacitive electrode design reduces the layer-by-layer manufacturing process by 20% (from five layers to four layers) and eliminates the need to align TX and RX electrodes during fabrication, enabling facile, scalable fabrication with improved uniformity in sensor-to-sensor variation and baseline capacitance variation. In conventional capacitive pressure sensor arrays, the capacitance formed between the sensing electrodes and a ground layer is typically not utilized as a sensing signal, while the ground layer primarily serves to suppress external electromagnetic interference (EMI). In contrast, our sensor design intentionally incorporates the electrode–GND capacitance into the pressure sensing mechanism. Under applied pressure, deformation of the dielectric and GND layers modulates this capacitance, enabling reliable detection of both static and dynamic loads. At the same time, the GND layers retain their role in shielding the sensor array from external EMI, ensuring stable operation and reliable real-time data acquisition. Demonstrations in healthcare and exercise monitoring applications, including sleep posture classification, pressure and musculoskeletal injury prevention, body balance assessment during weight training, and gait analysis, underscore the efficacy and versatility of the proposed large-area capacitive pressure sensor array (Fig. 1A).

**Fig. 1.**
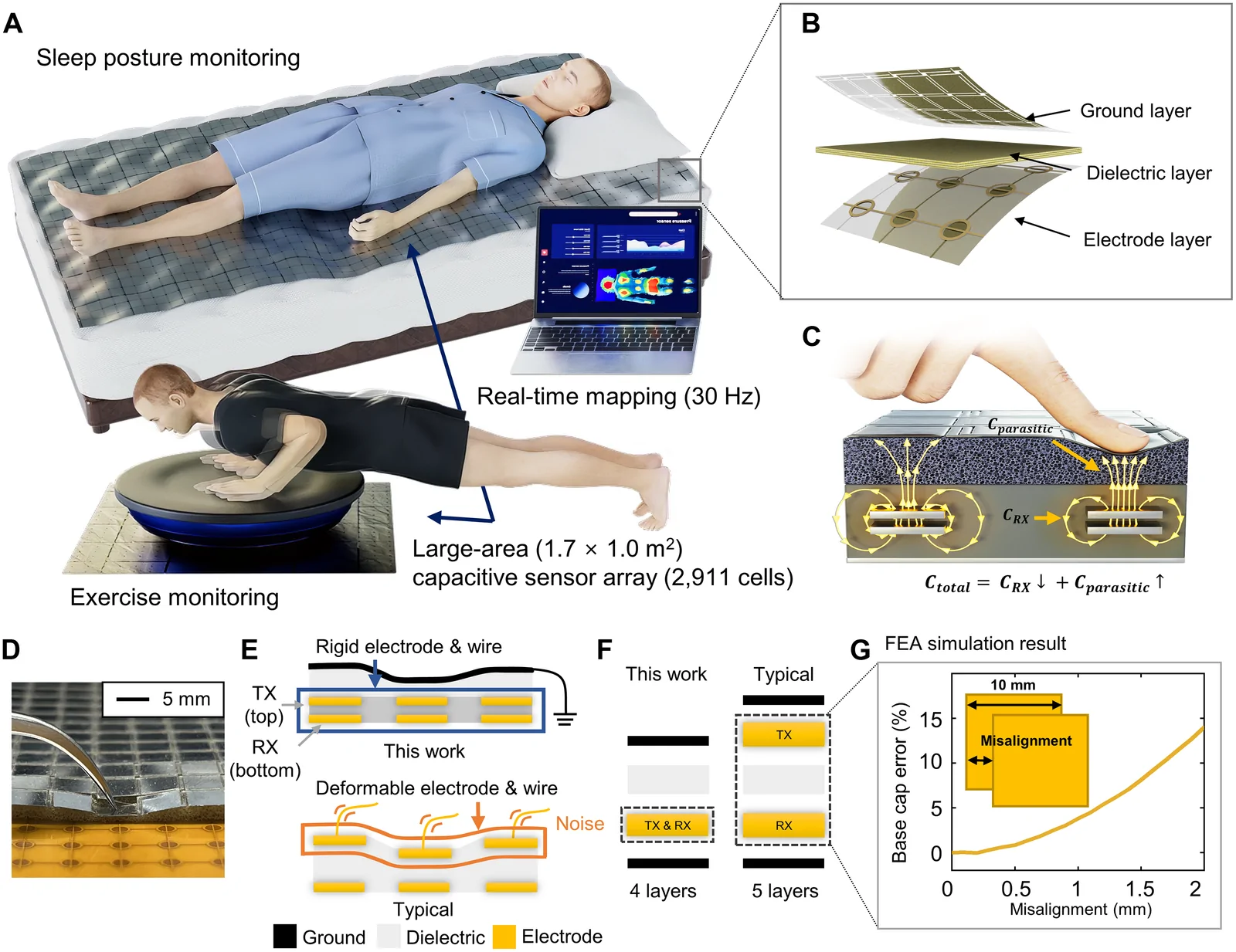
Overview of large-area pressure sensor array. (**A**) Healthcare applications and exercise monitoring using a large-area (1.7 m by 1.0 m) pressure sensor array capable of measuring static and dynamic pressure. (**B**) Exploded view of the pressure sensor array. (**C**) Working principle of the proposed sensor: a monolithic TX-RX electrode layer remains fixed while the dielectric and ground layers deform under applied pressure, resulting in changes in the mutual capacitance (*C*_RX_) and parasitic capacitance (*C*_parasitic_). (**D**) Photograph of the sensing cell under applied pressure. (**E**) The monolithic, fixed TX and RX electrodes, patterned on the top and bottom surfaces of the internal fPCB, respectively, eliminate signal noise from electrode movement and wired interconnection, which are typical challenges in conventional sensors. (**F**) The less stacked and simpler layer construction of the sensor design, enabled by the monolithic TX-RX architecture. (**G**) FEA simulation results of baseline capacitance errors caused by misalignment of TX and RX electrode layers in conventional sensors.

## RESULTS

### Large-area pressure sensor array and mechanism

The proposed sensor array system features a bottom electrode layer, in which TX and RX electrodes are firmly embedded on the top and bottom surfaces of a single fPCB, respectively (Figs. 1E and 2A). This innovative TX-RX architecture serves as the foundation for sequentially stacking a deformable dielectric layer (polyurethane foam) and a rationally designed top ground layer, as illustrated in Fig. 1B. The top ground layer plays a dual role by suppressing external capacitive coupling and approaching the fixed electrodes under applied pressure, thereby increasing the internal parasitic capacitance of the sensor for pressure sensing. In conventional capacitive sensors, parasitic capacitance induced by external EMI (for example, the human body or nearby electronics) is treated as noise and is removed (*22*). In our system, the ground layers block this external coupling, while internal parasitic capacitance of the sensor is intentionally exploited for pressure sensing, enabling a less stacked, simpler layer construction as shown in Fig. 1C, where total capacitance (*C*_total_) equals the sum of the mutual capacitance (*C*_RX_) formed between TX and RX and the parasitic capacitance (*C*_parasitic_) formed between TX and GND, as expressed in Eq. (1).

$$C_{\text{tota}\text{l}}=C_{\mathrm{RX}}+C_{\text{parasitic}}$$ (1)

**Fig. 2.**
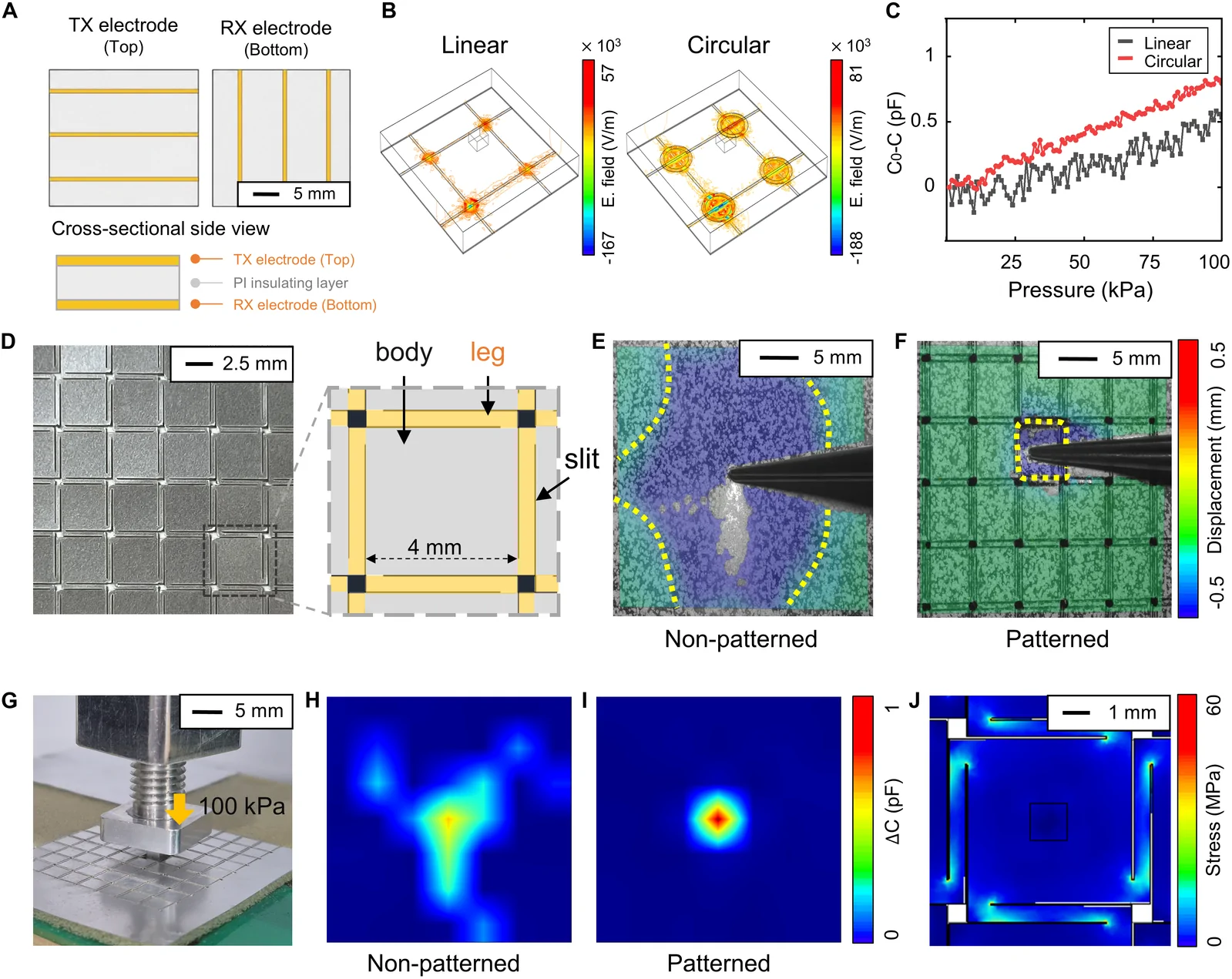
Design features of pressure sensor array. (**A**) The linear patterns (TX on the top surface and RX on the bottom surface of the fPCB) were designed as monolithic TX-RX electrode layers for projected capacitive (PCAP) sensors. (**B**) FEA simulation results of the electric field distribution with respect to the linear and circular patterns. (**C**) Changes in capacitance of the pressure sensors with linear and circular patterned electrodes. The circular pattern showed approximately 2.23 times better SNR (7.57) than the linear pattern (3.40). (**D**) The top GND layer, rationally patterned aluminum-deposited polyethylene terephthalate (Al-PET) film using a UV laser (body, 4 mm by 4 mm; four legs 4 mm by 0.5 mm). Digital image correlation (DIC) images of (**E**) redundant and large wrinkles on the non-patterned GND layer under pressure, and (**F**) the patterned GND layer under pressure. (**G**) Photograph of the 7 by 7 pressure sensor array under application of 100 kPa pressure, and the corresponding pressure mapping data for (**H**) the non-patterned GND layer and (**I**) the patterned GND layer. (**J**) FEA simulation results showing the stress distribution of the patterned GND layer under a pressure of 100 kPa.

As pressure is applied to the top of the GND layer in Fig. 1D and fig. S1, the top GND layer moves closer to TX electrodes, increasing *C*_parasitic_, while decreasing *C*_RX_ due to the change of electric field caused by the parasitic capacitance change (Fig. 1C). The readout IC therefore registers the applied pressure directly as this decrease in the TX–RX mutual capacitance. Figure 1, E to G present the superior advantages of our innovative sensor design, in which TX and RX electrodes are firmly embedded in the electrode layer like a touch screen, in terms of signal stability and manufacturing efficiency compared to conventional parallel-plate capacitive pressure sensors. In Fig. 1E, the fixed, monolithic TX-RX architecture of the sensor array system can effectively suppress signal noise caused by the movement of deformable electrode layers and wired interconnections linked to the multiplexer. On the other hand, the electrodes of conventional parallel-plate capacitive pressure sensors deform along with the exposed wired interconnections under applied pressure, thereby inducing unstable electrical connections, signal noise, and varying baseline capacitance in practical applications. Figure 1F illustrates the sensor structure consisting of four laminated layers. (i) a monolithic electrode layer including both fixed TX and RX electrodes, (ii) a soft dielectric layer composed of porous elastomer that is placed above the electrode layer, and (iii) two EMI shielding layers covering the entire assembly. In contrast, the conventional parallel-plate configuration typically requires five or more stacked layers, including separate TX and RX electrode layers. Above all, the less stacked and simpler layer construction of the sensor array, enabled by the monolithic TX-RX architecture, can reduce the layer-by-layer manufacturing process by 20% (from five layers to four layers) and eliminate the need to align TX and RX electrodes. These are significant advantages in the fabrication of large-area pressure mattresses for industrial applications, considering cost savings, scalable manufacturing, and improved uniformity in sensor-to-sensor variation and baseline capacitance variation (*23*). Figure 1G indicates FEA simulation results of baseline capacitance errors caused by misalignment of TX and RX electrode layers in conventional parallel-plate capacitive pressure sensors. As the misalignment of TX and RX electrode layers increases from 0 mm to 1 mm (10% of the initial dimension), the baseline capacitance error significantly increases by 5%. In particular, the nonlinear relationship between the misalignment and baseline capacitance error results in a gradually increasing error as the misalignment increases, leading to non-uniformity across sensing cells and unreliable data acquisition. These results were obtained from a simple model of two 10 mm by 10 mm square plates separated by a 2 mm dielectric, with equal x and y offsets and the error referenced to the perfectly aligned case.

### Design features of pressure sensor array

Electric fields are formed between the patterned TX and RX electrodes, and the direction and magnitude of the electric field can be modulated depending on the electrode design. The TX and RX electrodes, patterned on the top and bottom surfaces of the monolithic fPCB electrode layer at the bottom, play a significant role in determining the performance of the pressure sensor, as shown in Fig. 2A and fig. S2. To improve the sensitivity of the pressure sensor, circular patterns were adopted for TX and RX electrode design, as they enhance the projection of the electric field compared to the linear and rectangular patterns that are commonly used in projected capacitive (PCAP) touch screens (*24*). In Fig. 2B, FEA simulation results of four sensing cell configurations demonstrated that the circular pattern enhanced the magnitude of the electric field by 20%, indicating an increase from 2.24 V/m to 2.69 V/m compared to the linear pattern. Crosstalk in a capacitive pressure sensor array refers to unwanted signal interference, in which a pressure applied at a specific location generates responses in adjacent sensor cells, resulting in misleading readings during data acquisition. Figures S3 and S4 show FEA simulation results of cross-talk between sensing cells under applied pressure for linear, rectangular, and circular patterns. With respect to the evaluation for cross-talk in fig. S3, both linear and circular patterns exhibit lower but stable sensing signals (0.06 pF) from adjacent sensing cell under pressure compared to the rectangular pattern (0.48 pF), indicating that the circular pattern provides reliable and stable sensing signals with reduced cross-talk. In addition to this pattern-dependent cross-talk, a two-dimensional electrostatic simulation confirms that the electrical coupling between neighboring cells in the multiplexed array is negligible: with the grounded top plane, the charge induced on an adjacent cell is orders of magnitude below the addressed-cell signal and decreases rapidly with cell spacing (fig. S5). To evaluate the sensitivity (i.e., changes in capacitance under applied pressure of 100 kPa) of the pressure sensor according to the TX–RX pattern design of the electrode layer, the sensor was assembled sequentially (See Method Section for more details on the fabrication process). In Fig. 2C, the electrode layer with circular patterns exhibits 1.4 times higher sensitivity compared to the electrode layer with linear patterns as well as high linearity of 0.99, thanks to the enhanced electric field by 1.2 times according to the FEA simulation results in Fig. 2B. The FEA simulation results for the sensitivity of TX–RX pattern design in fig. S6 follow the trends of the experimental result in Fig. 2C. In addition, the comparatively small baseline capacitance of the electrode layer with linear patterns (2.44 pF) reduces the effective charge that can be detected and makes the sensing signal more vulnerable to parasitic capacitance and EMI, thereby degrading the signal-to-noise ratio compared to the electrode with circular patterns (6.61 pF), as shown in fig. S7. The sensor design with circular-patterned electrodes having a diameter (D) of 2 mm provides reliable sensing signals in terms of sensitivity, cross-talk, linearity, and high signal-to-noise ratio (SNR) value as shown in fig. S8. The SNR value of the circular pattern is 7.57, which is two times larger than the SNR value of the linear pattern, which is 3.4.

To improve the resolution of the sensor array, the top GND layer made of aluminum-deposited polyethylene terephthalate (Al-PET) film, was rationally patterned using a UV laser as shown in Fig. 2D. Al-PET film is an electrically conductive, cost-effective and mechanically robust material suitable for mass production of the pressure sensor array (table S1). Thanks to the structural patterning of the top GND layer (body, 4 mm by 4 mm; four legs 4 mm by 0.5 mm) by laser cutting, the pressure sensor array can accurately measure pressure distribution without causing any redundant or large wrinkles on the top GND layer under pressure, while maintaining electrical connectivity across the patterned structure. In Fig. 2, E and F, the area vertically displaced under applied pressure using a tweezer was observed using the digital image correlation (DIC) technique and highlighted with a yellow dotted line for non-patterned GND layer (60 mm^2^) and the patterned GND layer (16 mm^2^), respectively. The non-patterned GND layer in Fig. 2E exhibits a more wrinkled morphology (highlighted with a yellow dotted line) than the patterned GND layer under pressure in Fig. 2F, indicating that the rationally designed patterns provide a capacitive pressure sensor array with improved resolution. Likewise, fig. S9 presents DIC images of the GND layers under finger press, demonstrating the significance of the pattern design on the top GND layer. In addition, the continuous linkage of the top GND patterns through four legs across the sensor array ensures the electrical connection of GND layer, which is a significant factor for EMI shielding. Figure 2G shows the application of 100 kPa pressure to the pressure sensor array using a universal testing machine (UTM), and Fig. 2, H and I present the corresponding pressure mapping data for the applied pressure of 100 kPa. The pressure sensor with a non-patterned GND layer provides widespread pressure mapping data in Fig. 2H due to the wrinkle formation of the GND layer under applied pressure (peak ≈ 0.6 pF). In contrast, the pressure sensor with a patterned GND layer shows localized pressure mapping data under the same pressure of 100 kPa in Fig. 2I, presenting larger changes in capacitance (1 pF) and high resolution due to the patterned GND layer. The rationally designed patterns on the top GND layer contributed to the improved resolution of the sensor array and reduced wrinkle-induced mechanical cell-to-cell cross-talk. To further evaluate the cell-level response of the patterned GND layer, localized pressure was applied at the edge and corner positions within a single cell, rather than at the center only in fig. S10. When pressure was applied 1 mm diagonally offset from the center, the sensor response was nearly identical to the response obtained when pressure was applied at the center, demonstrating that the sensor response is robust to small positional variations within a cell. In contrast, when pressure was applied at the corner of a cell in fig. S11 (i.e., the inter-cell boundary), the sensor response was distributed across the four adjacent cells, which is attributed to mechanical coupling at the cell boundary. However, this response simultaneously provides additional spatial information that enables the perception of pressure localized at the corner. This inter-cell coupling effect is expected to be further alleviated in future work by reducing the TX–RX electrode pitch and the size of the laser-patterned GND electrodes, which would enhance the spatial resolution of the sensing array and minimize inter-cell physical cross-talk at boundaries. In addition, FEA simulations show the von Mises stress distribution of the Al-PET film under an applied pressure of 100 kPa, with a maximum stress below 60 MPa in Fig. 2G. The corresponding strain distribution is provided in fig. S12. This result defines the practical deformation range of the top GND layers during operation. In addition, FEA simulation results show the stress distribution of the Al-PET film under an applied pressure of 100 kPa, presenting a maximum stress of 60 MPa in Fig. 2J and fig. S12. This result provides the elastic deformation ranges of the top GND layers in practical applications, considering the yield stress of the Al-PET film (79 MPa).

### Performance characteristics of pressure sensor using parasitic capacitance

Figure 3A presents the changes in capacitance (C_0_-C) of the pressure sensor under loading and unloading at 120 kPa, given that the healthcare applications and the exercise monitoring require both low levels of pressure (0–5 kPa) and high levels of pressure (>100 kPa) (*4*–*6*, *25*–*27*). The results exhibited high linearity with an R^2^ value of 0.994 and a hysteresis of 3.3%. An electrostatic field simulation that includes only the parasitic coupling to the descending top ground plane reproduces this measured linear ΔC–pressure response (R^2^ = 0.994), confirming that the parasitic coupling is the actual transduction mechanism (fig. S13). Figure 3B presents the responses of ten individual sensing cells under an applied pressure of 30 kPa, with standard deviation values ranging from 0.98% to 1.59%. Figure 3C presents the response of the pressure sensor against three cyclic loadings of 50, 65, and 80 kPa, respectively. The results demonstrate stable and repeatable responses with clear discrimination among the three pressure levels and negligible baseline drift across cycles, which are significant characteristics for exercise monitoring. Figure 3D shows the changes in capacitance of the pressure sensor as the applied pressure increases in 0.25 kPa increments from 0 kPa to 1.5 kPa, indicating that the pressure sensor is capable of detecting subtle changes in pressure. Using the standard convention LOD = μ + 3σ, the shielded single-cell baseline noise (σ ≈ 0.4 fF) corresponds to a limit of detection of approximately 0.1 kPa (about 100 Pa) at the single-cell sensitivity of ≈ 11.3 fF/kPa (Fig. 3D). Figure 3E indicates the responses of the pressure sensor under 10,000 cyclic loadings of 30 kPa, demonstrating its durability and stable performance of the pressure sensor without drift. To minimize the environmental factors (e.g., external conductors, human skin, and EMI) that hamper accurate measurement of the applied pressure, the conductive GND layers on top and bottom sides were assembled with dielectric and electrode layers as shielding layers, as shown in Figs. 1B and 3F. In sequence, signal noise caused by EMI was measured by hovering various conductive objects, such as monkey spanner, screwdriver, and tweezer, over the pressure sensor, as shown in fig. S14 and movie S1. Thanks to both the top and bottom GND layers of the pressure sensor, stable signals could be obtained with the shielding layers. On the other hand, a noise of 0.08 pF, equivalent to the sensing signal for 5 kPa pressure, was observed without the shielding layers when a conductive object passed. Moreover, the change in capacitance caused by the noise from a metal jig was measured using UTM, as illustrated in Fig. 3G. The shielded pressure sensor maintained a stable baseline capacitance as the metal jig approached the pressure sensor and showed an increase in capacitance only after the application of pressure. However, the non-shielded pressure sensor showed a gradual increase in baseline capacitance as the metal jig approached, with the changes in capacitance of 0.1 pF prior to the application of pressure. Likewise, Fig. 3H further demonstrates the stability of the shielded pressure sensor under cyclic approaching and retracting motions of the metal jig using UTM. The shielded pressure sensor exhibited consistent and stable baseline capacitance, while the non-shielded pressure sensor exhibited repeated baseline shifts and significant fluctuations of 0.15 pF during each approach-retract cycle. Thus, the integration of the GND layers at the top and bottom sides of the pressure sensor for EMI shielding is indispensable for reliable and stable data acquisition in practical applications. To validate the versatility of the proposed sensing principle based on the parasitic capacitance with various dielectric materials, we conducted the experiment. The 3 by 3 TX–RX electrode array was additionally covered with three representative polymeric foams, such as polyurethane (PU), polyvinyl alcohol (PVA), and polyethylene (PE) as shown in fig. S15. The sensors exhibited sensitivities of 1.13 × 10^−2^ pF/kPa (PU), 0.53 × 10^−2^ pF/kPa (PVA), and 1.35 × 10^−2^ pF/kPa (PE) under an applied pressure of 100 kPa, with high linearity (R^2^) of 0.97, 0.93, and 0.95, respectively. Although PU foam was selected for the sensing platform owing to its high compressibility (large compression strain and low modulus) and low cost (table S2), these results demonstrate that the sensing mechanism is not restricted to a specific dielectric material but is applicable to various polymeric materials, subject to the manufacturing procedure and conditions. In addition, the patterned structure of the GND layers could generate slit-shaped openings under pressure loading. However, EMI from the outer environment (e.g., radio broadcast, RFID, BLE, Wi-Fi, and 5G) passing through the slit-shaped openings remains negligible, in accordance with the slit shielding theory (*28*–*30*). Here, shielding effectiveness (SE) represents how effectively the EMI shielding layer reduces the strength of interfering electromagnetic waves (e.g., radio broadcast, RFID, BLE, Wi-Fi, and 5G) passing through the pressure sensor in quantitative terms. A higher shielding effectiveness indicates better shielding from the outer environment. SE through slit-shaped openings can be expressed by Eq. (2) (*29*).

$$\text{SE}\approx 20{\text{log}}_{10}\left(\frac{\lambda }{2g}\right)$$ (2)

where λ is the wavelength of the electromagnetic wave, and g is the slit width under the pressure loadings of 100 kPa (gap width g≈1.2 mm). The shielding effectiveness at a frequency of 5 GHz (λ≈60 mm) is estimated as 28 dB shown in Eq. (3).

$$\text{SE}\approx 20{\text{log}}_{10}\left(\frac{60}{2\times 1.2}\right)\approx 28\text{dB}$$ (3)

**Fig. 3.**
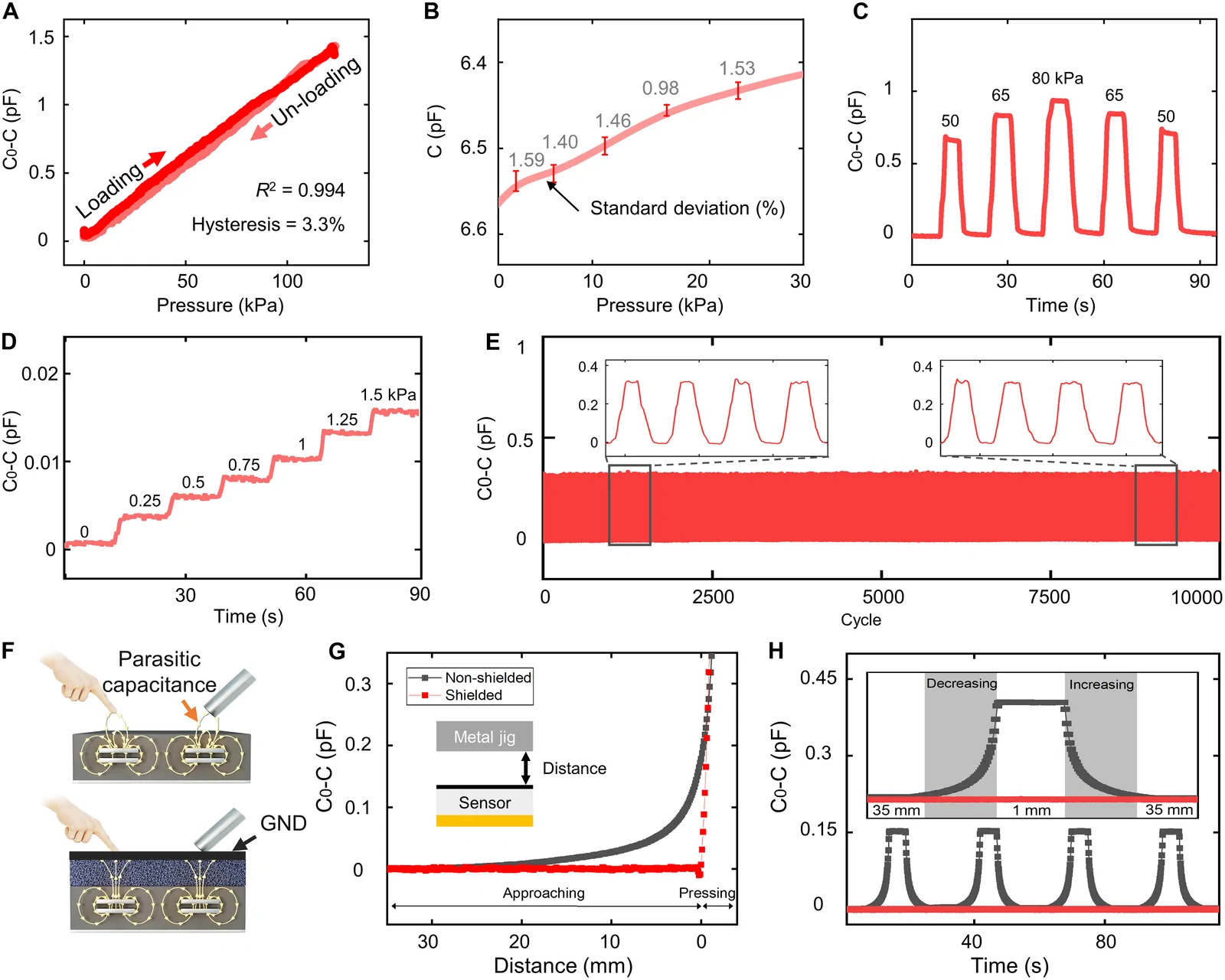
Performance characteristics of pressure sensor using parasitic capacitance. (**A**) The changes in capacitance (C_0_-C) of the pressure sensor under loading and unloading at 120 kPa. (**B**) Responses of ten individual sensing cells under an applied pressure of 30 kPa, with standard deviation values ranging from 0.98% to 1.59%. (**C**) Response of the pressure sensor against three cyclic loadings of 50, 65, and 80 kPa, respectively. (**D**) Response of the pressure sensor as an applied pressure increases by 0.25 kPa increments. (**E**) Response of the pressure sensor under 10,000 cyclic loadings of 30 kPa. (**F**) The schematic illustration of the shielding layers (top and bottom GND layers) that block interference from external conductive objects, ensuring reliable data acquisition. (**G**) Signal noise measured by hovering various conductive objects, such as monkey spanner, screwdriver, and tweezer, over the pressure sensor. (**H**) Signal noise measured under cyclic approaching and retracting motions using UTM.

As a shielding effectiveness of 20 dB or higher is regarded as sufficient for everyday electromagnetic interference (EMI) mitigation, the patterned structure of the GND layers could provide robust EMI shielding in practical scenarios (table S3).

### Large-area pressure sensor array and implementation of module system

Based on the innovative, scalable structural characteristics of the pressure sensor, such as the monolithic TX-RX electrode layer, the rationally patterned GND layers, and the dielectric layer in between, a large-area pressure sensor array that measures 1.7 m by 1.0 m using 2911 sensing cells (i.e., 41 by 71 sensor array) was developed for real-time measurement of the pressure distribution, as shown in Fig. 4A. The block diagram of the sensor array and sensing module system (i.e., readout module) is illustrated in Fig. 4B. The module system is designed to convert the large-area pressure distribution measured by the 2911 sensing cells into electrical signals and digital mapping data using a customized LabVIEW-based software at a sampling frequency of 30 Hz. The module system is based on the microcontroller and incorporates the touchscreen controller for precise capacitance measurement. The overall system operates with low power consumption (80 mA at 5 V), integrated with High-speed SPI and UART interfaces for real-time visualization mapping data from the touchscreen controller. For the protection of subjects exposed to radiofrequency electromagnetic fields (RF EMF), we measured the electromagnetic field strength generated by the sensor array system during operation using a commercial electromagnetic field meter (KDsafe, South Korea) in Fig. 4C. The electromagnetic field meter indicated that the emitted electromagnetic field strength of 74 V/m (electric field) and 0.12 μT (magnetic field) remained within the RF EMF guidelines of the International Commission on Non-Ionizing Radiation Protection (ICNIRP) for 100–1000 kHz range (table S4) (*31*). This is because the GND layer not only shields signal noise from external conductors, but also shields the electromagnetic field from the inside electrode layers so that it is not emitted outside. In Fig. 4D, furthermore, when measuring gait pressure during fast walking, the sensor array demonstrated a quick response, aligning with the pace of foot movement. This is due to the rapid data acquisition of the touchscreen integrated circuit (IC) when applying dynamic pressure, which collected 30 frames per second. This reliable and rapid pressure mapping data acquisition for large-area can be applied to diverse healthcare application fields that require dynamic and fast load tracking (e.g., exercise monitoring, sports and entertainment). In addition, Fig. 4E and movie S2 demonstrate the excellent high resolution of the large-area pressure sensor array, which can distinguish the millimeter-scale variations, as four objects with a difference of 5 mm in diameter (25, 30, 35, and 40 mm) are shown in the pressure mapping data. The result clearly indicates that the sensor array is capable of accurately recognizing small differences in object size (5 mm variations), highlighting its great potential for biomedical and industrial applications, such as robotic hands and rehabilitation, where replicating the human ability to perceive two points on the skin (i.e., two-point threshold) is significant (*32*).

**Fig. 4.**
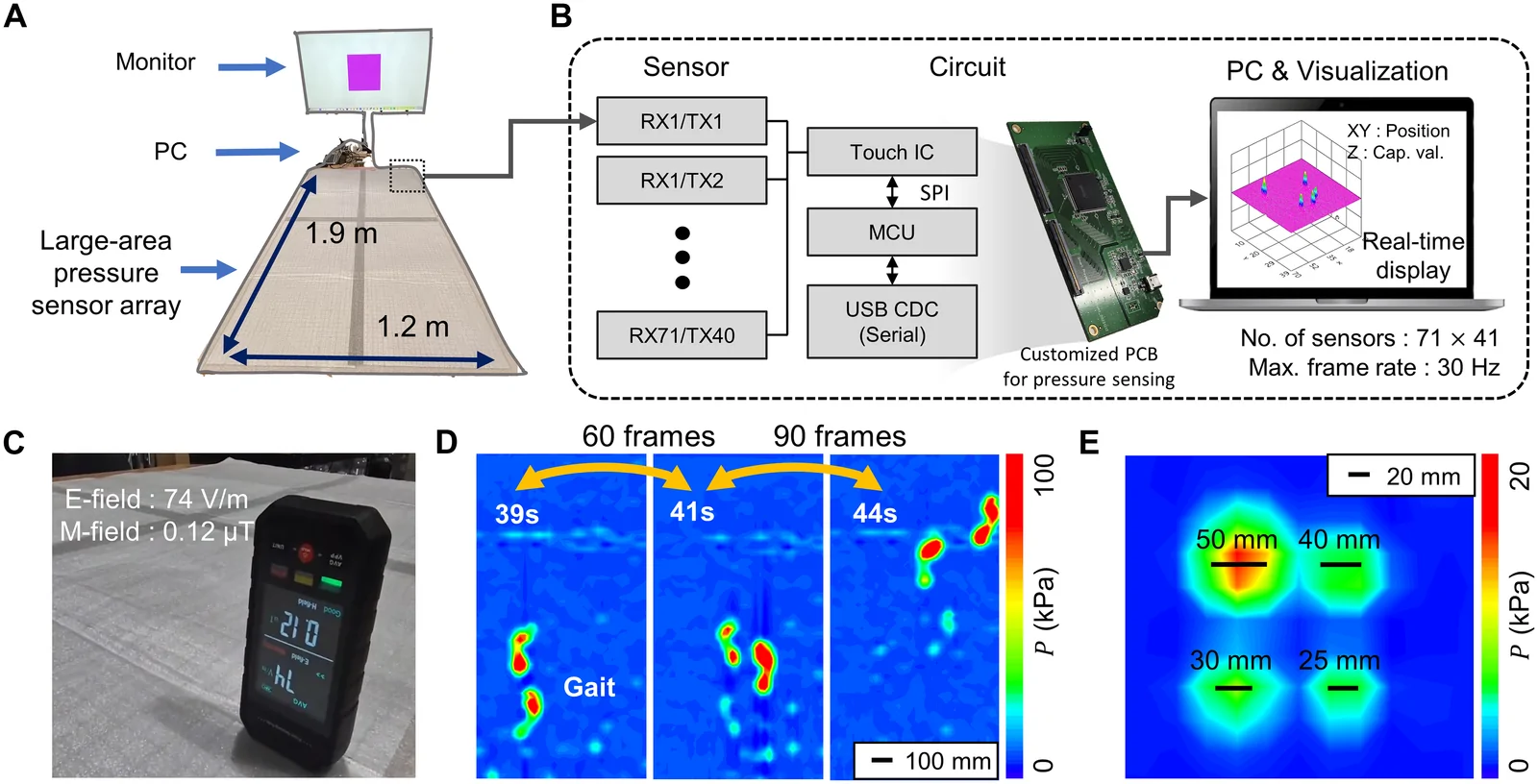
Features of large-area sensor array and implementation of module system. (**A**) Photograph of large-area pressure sensor array that measures 1.7 m by 1.0 m using 2911 sensing cells (41 by 71 sensor array), connected to a module system and PC for real-time pressure distribution measurement. (**B**) Block diagram of the sensor array and sensing module system to convert the large-area pressure distribution measured by the 2911 sensing cells into electrical signals and digital mapping data at a high speed of 30 Hz. (**C**) Electromagnetic field strength measurement during operation using a commercial electromagnetic field meter. (**D**) Real-time rapid data acquisition (30 frames/s) of the pressure sensor array during fast walking (gait analysis). Pressure distribution changes are visualized, with clear detection of foot contact points at different time intervals (39 s, 41 s, and 44 s). (**E**) High resolution performance of the large-area pressure sensor array that can distinguish the millimeter-scale variations [i.e., four objects with a difference of 5 mm in diameter (25, 30, 35, and 40 mm)].

### Application of large-area pressure sensor array for healthcare applications

Pressure injuries develop in the skin and underlying tissues around bony prominences due to the prolonged pressure at skin interfaces. In other words, when pressure is applied for prolonged periods, deformation of the skin tissue hinders blood circulation and restricts oxygen and nutrient supply to the tissue, leading to tissue necrosis (*33*, *34*). In particular, elderly patients with limited mobility (e.g., sleeping, paralyzed, and bed-confined) are prone to pressure injuries in hospitals (*35*, *36*). As the development of pressure injuries has a substantial impact on patient’s quality of life and imposes huge economic burdens for treatment and therapy (*37*), efforts to prevent pressure injuries have been addressed by using sensor array systems in accordance with repositioning guidelines (*8*, *38*, *39*). Despite the importance of the pressure sensor arrays, discriminating exact postures and mapping precise areas under pressure demand substantial effort due to the difficulties in integrating multiple technologies, such as rapid and reliable data acquisition, scalable sensor design, excellent sensor characteristics (e.g., sensitivity, linearity, resolution, durability), uniformity (i.e., sensor-to-sensor variation), module system design, and advanced post-processing (e.g., machine learning, and temperature/humidity compensation) (*10*, *19*, *20*, *40*–*42*). Herein, we integrated the large-area pressure sensor array with a commercial alternating air pressure mattress for an automated pressure mapping system based on real-time measurement of pressure distribution through excellent sensor characteristics. The alternating air pressure mattress operates in two states (i.e., inflation (state 1) and deflation (state 2) of air cells) for the prevention of pressure injuries, as shown in Fig. 5A and movie S3. Figure 5B presents the pressure distribution data acquired for each state of the alternating air pressure mattress. Our high-resolution pressure sensor array enables accurate identification of the areas under load while the patient maintains the same posture, and the areas under load vary as the alternating air pressure mattress alternates between state 1 and state 2. In addition to monitoring posture for repositioning of bed-confined patients at regular intervals to prevent pressure injuries, posture monitoring can also be applied to healthcare applications, such as the management of musculoskeletal injuries (e.g., the prone position is a bad posture for disc herniation) and sleep posture monitoring to assess sleep quality by checking the frequency of posture changes. Figure 5, C and D present a subject (28-year-old; male; height, 178 cm; mass, 85 kg) lying in the bed in four different sleep postures, such as supine, prone, left lateral, and right lateral postures, and the corresponding pressure mapping data, respectively. A compact two-dimensional convolutional neural network (2D CNN) was used to classify the four postures directly from the 41 by 71 pressure maps. As shown in Fig. 5E, the network consists of three convolutional blocks (with MaxPool 2 by 2 in the first two blocks) followed by a global average pooling layer and a small dense head, which classifies each input into one of four posture classes (supine, prone, left lateral, right lateral). See the Materials and Methods for detailed information on the network architecture and training protocol. To evaluate generalization to unseen individuals, five-subject leave-one-subject-out (LOSO) cross-validation was performed (subjects: age 26–31 y, height 174–178 cm, weight 75–85 kg, BMI 24.8–26.8). Across the five LOSO folds (48,005 test predictions in total), the aggregated confusion matrix in Fig. 5F is essentially diagonal, yielding a mean accuracy of 97.41 ± 5.67% (macro F1 = 0.981). These results demonstrate that the proposed pressure sensor array-based monitoring system can classify sleeping postures with high confidence even for previously unseen individuals. Compared to the previous monitoring systems, including vision-based monitoring, pressure sensor array-based monitoring systems offer several advantages. In particular, a pressure sensor array provides complete privacy for users. Since the physical sensing system does not capture any visual images, users can sleep without concerns about privacy invasion. In addition, the precise measurement of pressure distribution using the sensor array enables precision healthcare applications in hospitals, allowing medical doctors to refer to information on posture, frequency of posture changes, and sleep patterns for long-term monitoring. The findings of this study demonstrate the potential of pressure sensor array-based monitoring system not only for the prevention of pressure injuries, but also the analysis of sleep patterns, the diagnosis of sleep disorders, and the treatment to improve sleep quality.

**Fig. 5.**
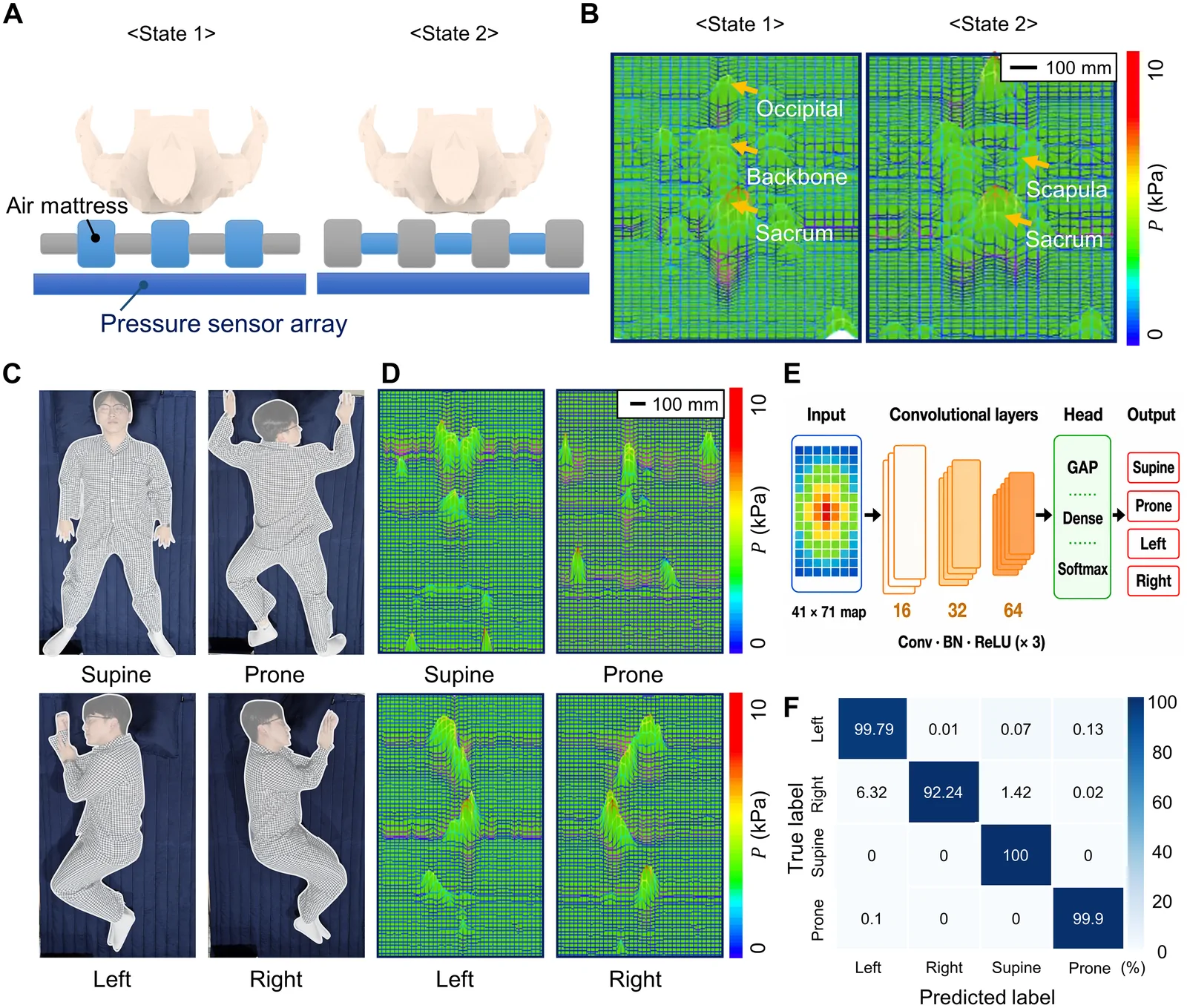
Healthcare applications using large-area sensor array. (**A**) Schematic illustration of the pressure sensor array integrated with an alternating air pressure mattress for an automated pressure-relieving system based on real-time measurement of pressure distribution. (**B**) Pressure distribution data acquired for each state of the alternating air pressure mattress operating in two states (i.e., inflation and deflation of air cells). (**C**) Photographs of a subject (28-year-old; male; height, 178 cm; mass, 85 kg) lying in the bed in four different sleep postures, such as supine, prone, left lateral, and right lateral postures, and (**D**) the corresponding pressure mapping data. (**E**) A compact two-dimensional convolutional neural network (2D CNN) processes the 41 by 71 pressure map, featuring three convolutional blocks (with MaxPool in the first two), a global average pooling layer, and a small dense head that classifies the input into one of four posture classes (supine, prone, left lateral, right lateral). (**F**) Aggregated confusion matrix over the five leave-one-subject-out (LOSO) folds (48,005 test predictions), yielding a mean accuracy of 97.41 ± 5.67% (macro F1 = 0.981).

### Application of large-area pressure sensor array for exercise monitoring

Thanks to the immediate and quantitative responses of the pressure sensor during exercise, pressure sensors have been developed for exercise monitoring (*7*, *25*, *43*). Among the various pressure sensor types, capacitive sensors are suitable for exercise monitoring because capacitive pressure sensors offer excellent performance characteristics (i.e., fast response, high sensitivity, linearity, and low drift) without the need for compensation for humidity and temperature during operation. However, difficulties, such as large-scale manufacturing, array configurations, and signal noise from external factors have limited their practical use in exercise monitoring. Nonetheless, the pressure sensor array developed in this study addresses these challenges through the advantages of the innovative capacitive sensing method, facile fabrication of array configurations, and signal noise reduction via EMI shielding, making our system highly suitable for exercise monitoring applications (movie S4).

In Fig. 6A, as the subject performs squats while shifting their weight from backward to forward, changes in pressure distribution are observed, enabling the trainer to teach the correct squat posture based on the pressure mapping data (e.g., body balance, movement of the center of weight). Likewise, during sit-ups, changes in pressure distribution are noticeable between the correct posture and two incorrect postures (i.e., right and left twisted), as shown in Fig. 6B. Moreover, the instrumentation circuit, which rapidly scans 2911 cells of the pressure sensor array at 30 Hz, enables the monitoring of subtle body movements and provides detailed, quantitative information on instantaneous body imbalance.

**Fig. 6.**
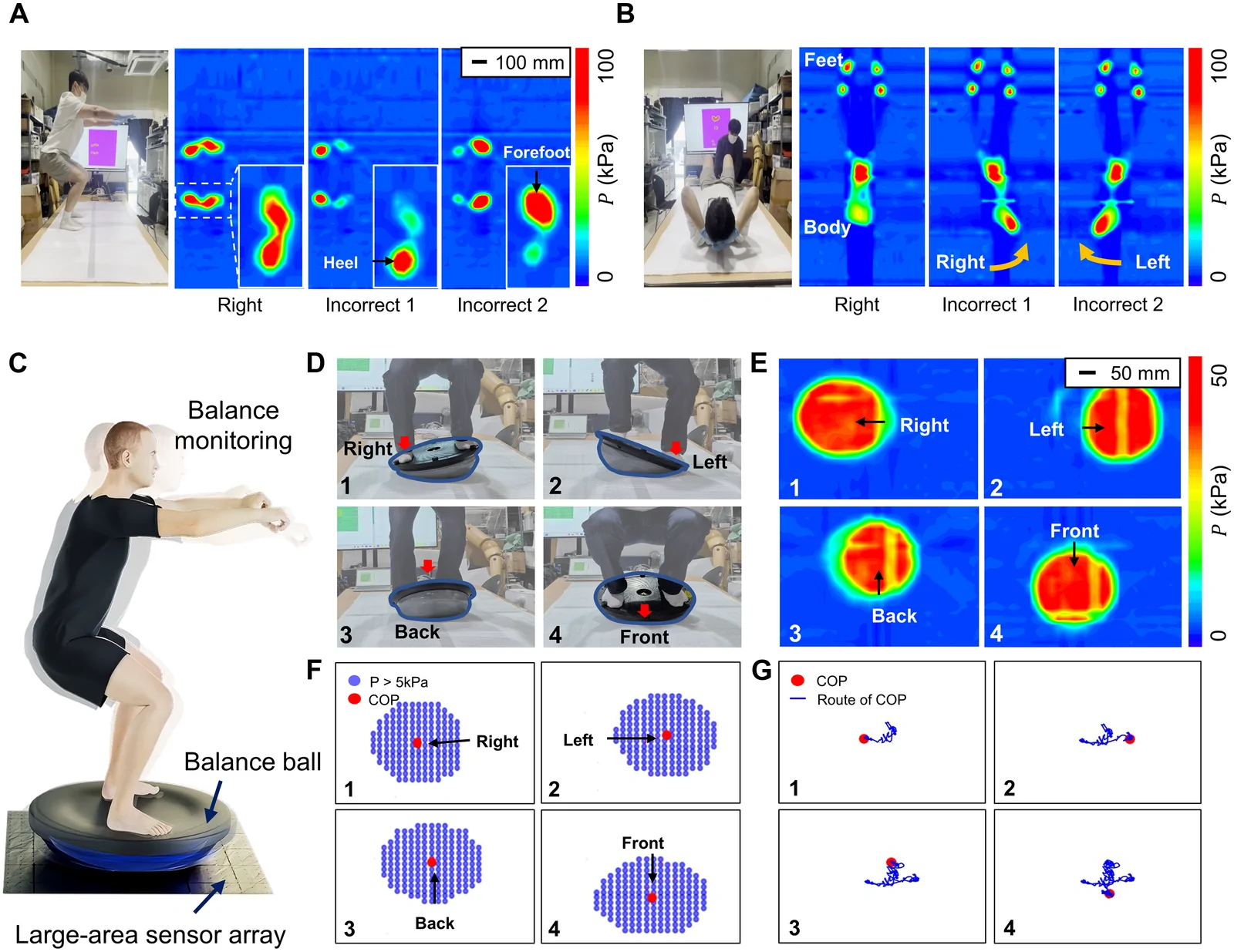
Exercise monitoring using large-area sensor array. The high-speed sensor array system, capable of scanning 2911 sensing cells at 30 Hz, enables real-time exercise monitoring. The subject performing (**A**) squats while shifting their weight from backward to forward, with corresponding pressure mapping data, and (**B**) sit-ups while twisting their posture from right to left, with corresponding pressure mapping data. (**C**) Schematic illustration of body balance monitoring system. The sensor array system integrated with a balance ball to monitor body balance and core stability during exercise. (**D**) Four primary imbalance postures, such as right, left, backward, and forward shifts, are identified based on (**E**) pressure mapping data for each imbalance posture. (**F**) Pressure mapping data highlight areas where pressure exceeds 5 kPa to visualize excessive pressure applied during exercise. (**G**) The center of pressure (COP) trajectory plot tracks weight shifts over time, illustrating movement patterns.

The use of balance balls in exercise is designed to enhance core strength and improve body balance. Therefore, the exercise monitoring system that tracks the movement of the center of weight would provide the users with quantitative data on body balance. Tracking the movement of the center of weight requires a sensor array system that rapidly scans numerous sensing cells, as shown in movie S5. In the proposed system, a balance ball is placed on top of the pressure sensor array and the subject performs exercise in Fig. 6C. During the exercise, the center of weight shifts from the right to the left and back and forth as shown in Fig. 6D. This sensor array system tracks and presents the locations where the center of pressure (COP) is applied (Fig. 6E). From the recorded data, sensing cells under pressure of more than 5 kPa were extracted, and COP was subsequently calculated based on these spatial coordinates (Fig. 6F). By displaying the COP pathway, our system helps users understand the center of weight movements during exercise, thereby gradually enhancing the core strength and improving body balance based on the quantitative mapping data. In addition, since exposure to sweat and moisture during exercise monitoring can affect the performance of the pressure sensor, the outer surface of the pressure mattress was covered with a hydrophobic polyethylene (PE) foam protective layer (fig. S16, A and B). The protective layer effectively prevented liquid penetration into the underlying electrode and dielectric layers. A 3 by 3 array of the pressure sensor was continuously read out at 30 Hz while a water droplet was placed on top of the PE foam. Across the entire observation, the baseline capacitance of every cell remained within ±10 fF, showing no shift or drift even at the moment the droplet was placed (fig. S16, C and D). Owing to this protective layer, no performance degradation was observed during the validation trials, demonstrating the robustness of the sensor array against moisture exposure in practical exercise monitoring scenarios.

## DISCUSSION

This study presents monolithic TX-RX capacitive electrodes for a novel pressure sensor array that leverages parasitic capacitance for reliable, precise, and scalable full-body pressure mapping. The unique integration of TX-RX electrodes into a single, fixed substrate significantly enhances manufacturing efficiency by eliminating the electrode alignment process and reducing the multilayer stacking process by 20%, resulting in a facile fabrication process. Although parasitic capacitance is regarded as signal noise or negative interference in conventional pressure sensors, leveraging parasitic capacitance as a primary sensing parameter in this work enables reliable data acquisition by suppressing signal noise and improving sensitivity. Furthermore, the design of a rationally patterned top GND layer improves the resolution of the pressure sensor array through localized deformation at the millimeter scale and shields against EMI. Compared with iontronic capacitive sensors, which provide very large per-cell capacitance and intrinsic low-frequency EMI rejection and are therefore well suited to small-area, high-sensitivity wearables, the present picofarad-scale architecture is optimized for large-area, multi-thousand-cell arrays read by mass-produced mutual-capacitance controllers; a system-level comparison of the two approaches (table S5) shows that they are complementary rather than competing.

The developed large-area pressure sensor array, incorporating 2911 sensing cells over a 1.7 m by 1.0 m area, demonstrates high resolution pressure mapping at a fast scanning rate of 30 Hz, suitable for both static and dynamic sensing scenarios (i.e., healthcare applications and exercise monitoring). The outstanding performance characteristics of the pressure sensor array, including low hysteresis (3.3%), excellent linearity (R^2^ = 0.994), stable baseline capacitance, and robust EMI shielding, highlight its great potential for both industrial and precision healthcare applications. Experimental validations in healthcare applications such as pressure injury prevention, sleep posture classification using a 2D convolutional neural network, and exercise monitoring underscore the efficacy and versatility of the sensor array in diverse scenarios. Because the top ground layer is a structural prerequisite for any large-area TX/RX capacitive array—it shields external EMI and forms the electrical-cross-talk barrier—its mass is not removable overhead; in this design the same layer additionally transduces pressure, so one shared layer serves multiple functions and the four-layer stack reaches square-meter scale at low added weight.

Beyond the sensor architecture itself, the sleep posture classification demonstrated in this work relied on a compact 2D convolutional neural network evaluated with leave-one-subject-out cross-validation on five healthy adult subjects. While the present cohort is intentionally scoped to healthy adult male subjects (n = 5) to first establish the per-subject generalization of the proposed sensing architecture, future work will scale the dataset to broader demographic categories — including female subjects and a wider range of body habitus—to further sharpen and broaden the across-fold mean.

This work provides a significant advancement in capacitive pressure sensor technology, paving the way for its widespread adoption in healthcare, rehabilitation, exercise monitoring, robotics, and human-machine interfaces. Future research could explore further miniaturization, integration with IoT systems, and advanced data analytics techniques to expand the applications and enhance the functionality of the proposed sensing platform. Additionally, further studies on the design of the GND layer could enable the measurement of parameters beyond pressure (e.g., shear, torsion and strain), extending the multi-modality of the sensor. Development of dielectric materials at the micro and nano scale could enhance the scalability and performance characteristics of the platform. Moreover, comprehensive comparative studies on the design of TX-RX electrode patterns could optimize the spatial resolution and other key performances for broader applications. To demonstrate operation on non-planar surfaces, the sensor was characterized on cylindrical mandrels (R = 100, 60, and 40 mm); the pressed-cell response remains linear on all curved substrates and the per-cell sensitivity decreases with decreasing radius, so that singly-curved deployment is possible with a simple per-radius re-baselining (fig. S17).

## MATERIALS AND METHODS

### Materials

For the GND layer, a 100 μm thick PET film with aluminum deposition was used (table S1), purchased from SheetMeka (South Korea). The dielectric layer consisted of 2 mm-thick polyurethane foam (table S2). For the electrode layer, fPCB was initially used to evaluate the effects of electrode shape. For large-area sensor fabrication, a customizable touch foil was purchased from Greentouch (China) and employed accordingly.

### Finite element analysis

All finite element simulations were performed in COMSOL Multiphysics (electro-mechanical module). A parallel plate configuration was modeled with TX and RX plates separated by a uniform dielectric. Equal in-plane offsets were applied along *x* and *y* to emulate simultaneous misalignment, and capacitance was referenced to the perfectly aligned case. The baseline error increased nonlinearly with offset. A 10 percent misalignment resulted in a 5 percent baseline error, consistent with Fig. 1G. Parametric simulations were performed to evaluate the RX and TX electrode structures. The analysis focused on two performance metrics: cross-talk and sensitivity. Three electrode geometries—linear, circular, and rectangular—were modeled (figs. S2 and S3), along with variations in electrode size (fig. S4). The simulation conditions matched the physical dimensions and material properties of the fabricated sensors. To evaluate cross-talk, a 2 by 2 sensor array was constructed, and localized pressure was applied at the center cell to observe electric field propagation to adjacent elements (fig. S4). For sensitivity analysis, a single sensor cell was modeled, and the electric field change was recorded in response to localized pressure applied at the center (fig. S6).

### Characterization of pressure sensor

Following the comparative evaluation of electrode geometries, the circular layout was selected and sensor arrays were fabricated on a printed circuit board. Top and bottom ground shields were included as designed. Mechanical loading was applied using a universal testing machine (AGS-X, SHIMADZU, Japan). A square flat indenter of 3 mm by 3 mm was used to deliver localized pressure at the center of a sensing cell. Pressure was obtained from the measured force and the contact area. The force transducer provided ±0.5% readout accuracy with a 1 kN capacity. Capacitance was measured with a precision LCR meter (E4980A, Agilent, USA) at 400 kHz, and long duration or fast cycling was recorded with a CDC (PCap04, ScioSense, Netherlands). The test matrix covered linearity and hysteresis under ramp loading to 120 kPa and unloading, repeatability and cell-to-cell uniformity at fixed pressure, dynamic response under periodic loading, resolution using 0.25 kPa steps, durability for 10,000 cycles at 30 kPa, and cross-talk and parasitic coupling assessments using a grounded metal jig and approaching conductive tools. GND layer was connected to the instrument ground.

### Development of pressure sensor array controller

A custom capacitive pressure sensor controller was developed to support high-speed acquisition in large-area sensor arrays. The system manages a 41 by 71 array (2911 nodes) with a sampling rate of up to 30 Hz and a 10-bit resolution (0–1023 levels). It is built on the nRF52840-M4 microcontroller (Nordic, ARM Cortex-M4) and integrates the MXT2912 touch IC for precise capacitance measurement. Sensor data are transmitted to a PC via a USB-C interface (Type-C connector), and internal communication with the sensor array is achieved through fPCB connectors. The system operates with low power consumption, remaining under 80 mA at 5 V. Firmware development was based on Zephyr RTOS (NCS 2.2.0), enabling core functionalities such as USB CDC communication, touch IC control, and timer/LED management. High-speed SPI (8 MHz) and UART (57,600 bps) interfaces facilitate real-time data exchange with the touch IC. A flexible communication protocol supports data streaming, adjustable output rates (10, 15, 20, or 30 Hz), zero calibration, device reset, time logging, and data retrieval. The controller also supports multi-touch detection and cumulative usage time tracking, ensuring reliable performance for large-area pressure sensing applications.

### Pressure mapping procedure

The capacitance change (ΔC) at each cell of the sensor array was measured by the mutual-capacitance IC in a cell-by-cell, row-by-row sequential scanning manner at a 30 Hz frame rate. During each scan, one TX line is driven while the remaining TX lines are held at ground and all RX lines are read simultaneously, so that each cell is measured independently and unaddressed lines contribute no signal. Because the RX lines are read in parallel, the frame rate is set mainly by the number of TX lines and the time to measure one line, and can be increased by reading groups of TX lines with additional readout ICs in parallel. The scanned array data were then transferred to a custom LabVIEW routine, where row-wise linear interpolation was applied along each row to render the two-dimensional contour maps shown in Fig. 2, H and I.

### Two-dimensional convolutional neural network for sleeping posture classification

A compact two-dimensional convolutional neural network (2D CNN) was trained to classify sleeping postures directly from the 41 by 71 pressure map. Each frame was normalized by two steps — per-frame sum normalization and per-subject z-score standardization — before being fed to the network. The 2D CNN consisted of three convolutional blocks with 16, 32, and 64 filters (each followed by BatchNormalization and ReLU, and 2 by 2 MaxPooling in the first two blocks), Global Average Pooling, a small dense head with dropout, and a four-class softmax output (supine, prone, left lateral, and right lateral). The network was trained with sparse categorical cross-entropy loss and the Adam optimizer, with balanced class weights to compensate for class imbalance and early stopping on validation loss.

Generalization was evaluated with five-subject leave-one-subject-out (LOSO) cross-validation. Robustness to user displacement was further assessed by applying random translations of up to ±24 cm and rotations of up to ±20° to the test frames without retraining. Per-subject demographics and dataset statistics are provided in table S6, and figs. S18 and S19. The position-robustness results are provided in fig. S20 (all five folds) and table S7 (joint sweep across the four folds excluding S03). 

## References

[1] N. A. Abd Razak, N. A. Abu Osman, H. Gholizadeh, S. Ali, Biomechanics principle of elbow joint for transhumeral prostheses: Comparison of normal hand, body-powered, myoelectric & air splint prostheses. Biomed. Eng. Online 13, 134 (2014). 10.1186/1475-925X-13-134 PMC4228154 25208636

[2] A. Vitali, D. Regazzoni, C. Rizzi, G. Colombo, Design and additive manufacturing of lower limb prosthetic socket, in *ASME international mechanical engineering congress and exposition* (American Society of Mechanical Engineers, 2017), vol. 58462 V011T15A021.

[3] M. Han, L. Chen, K. Aras, C. Liang, X. Chen, H. Zhao, K. Li, N. R. Faye, B. Sun, J. H. Kim, W. Bai, Q. Yang, Y. Ma, W. Lu, E. Song, J. M. Baek, Y. Lee, C. Liu, J. B. Model, G. Yang, R. Ghaffari, Y. Huang, I. R. Efimov, J. A. Rogers, Catheter-integrated soft multilayer electronic arrays for multiplexed sensing and actuation during cardiac surgery. Nat. Biomed. Eng. 4, 997–1009 (2020). 10.1038/s41551-020-00604-w PMC8021456 32895515

[4] H. Han, Y. S. Oh, S. Cho, H. Park, S.-U. Lee, K. Ko, J.-M. Park, J. Choi, J.-H. Ha, C. Han, Z. Zhao, Z. Liu, Z. Xie, J.-S. Lee, W. G. Min, B.-J. Lee, J. Koo, D. Y. Choi, M. Je, J.-Y. Sun, I. Park, Battery-free, wireless, ionic liquid sensor arrays to monitor pressure and temperature of patients in bed and wheelchair. Small 19, e2205048 (2023). 10.1002/smll.202205048 36534830

[5] Y. S. Oh, J. H. Kim, Z. Xie, S. Cho, H. Han, S. W. Jeon, M. Park, M. Namkoong, R. Avila, Z. Song, S. U. Lee, K. Ko, J. Lee, J. S. Lee, W. G. Min, B. J. Lee, M. Choi, H. U. Chung, J. Kim, M. Han, J. Koo, Y. S. Choi, S. S. Kwak, S. B. Kim, J. Kim, J. Choi, C. M. Kang, J. U. Kim, K. Kwon, S. M. Won, J. M. Baek, Y. Lee, S. Y. Kim, W. Lu, A. Vazquez-Guardado, H. Jeong, H. Ryu, G. Lee, K. Kim, S. Kim, M. S. Kim, J. Choi, D. Y. Choi, Q. Yang, H. Zhao, W. Bai, H. Jang, Y. Yu, J. Lim, X. Guo, B. H. Kim, S. Jeon, C. Davies, A. Banks, H. J. Sung, Y. Huang, I. Park, J. A. Rogers, Battery-free, wireless soft sensors for continuous multi-site measurements of pressure and temperature from patients at risk for pressure injuries. Nat. Commun. 12, 5008 (2021). 10.1038/s41467-021-25324-w PMC8385057 34429436

[6] S. Cho, H. Han, H. Park, S.-U. Lee, J.-H. Kim, S. W. Jeon, M. Wang, R. Avila, Z. Xi, K. Ko, M. Park, J. Lee, M. Choi, J.-S. Lee, W. G. Min, B.-J. Lee, S. Lee, J. Choi, J. Gu, J. Park, M. S. Kim, J. Ahn, O. Gul, C. Han, G. Lee, S. Kim, K. Kim, J. Kim, C. M. Kang, J. Koo, S. S. Kwak, S. Kim, D. Y. Choi, S. Jeon, H. J. Sung, Y. B. Park, M. Je, Y. T. Cho, Y. S. Oh, I. Park, Wireless, multimodal sensors for continuous measurement of pressure, temperature, and hydration of patients in wheelchair. npj Flex. Electron. 7, 8 (2023).

[7] J. Choi, C. Han, D. Lee, H. Kim, G. Lee, J.-H. Ha, Y. Jeong, J. Ahn, H. Park, H. Han, S. Cho, J. Gu, I. Park, Thermoforming 2D films into 3D electronics for high-performance, customizable tactile sensing. Sci. Adv. 11, eadv0057 (2025). 10.1126/sciadv.adv0057 PMC12077495 40367155

[8] H. Han, H. Park, S. Cho, S. U. Lee, J. Choi, J. H. Ha, J. Park, Y. Jung, H. Kim, J. Ahn, Y. J. Kwon, Y. S. Oh, M. Je, I. Park, Battery-free, wireless multi-modal sensor, and actuator array system for pressure injury prevention. Small 20, 2405493 (2024). 10.1002/smll.202405493 39087376

[9] H. Han, H. Kim, S. Cho, D. Lee, J. Moon, C. Kim, J.-H. Ha, Y. Jung, Y. S. Oh, J. Choi, I. Park, Wireless, battery-free multi-axial sensor for augmented reality-assisted monitoring at skin interfaces. npj Flex. Electron. 9, 102 (2025).

[10] Z. Lin, J. Yang, X. Li, Y. Wu, W. Wei, J. Liu, J. Chen, J. Yang, Large-scale and washable smart textiles based on triboelectric nanogenerator arrays for self-powered sleeping monitoring. Adv. Funct. Mater. 28, 1704112 (2018).

[11] W.-Y. Chang, C.-C. Huang, C.-C. Chen, C.-C. Chang, C.-L. Yang, Design of a novel flexible capacitive sensing mattress for monitoring sleeping respiratory. Sensors 14, 22021–22038 (2014). 10.3390/s141122021 PMC4279575 25420152

[12] H. L. Tey, C. Z. Y. Phuan, J. S. T. Tan, S. Yang, N. O. Hnin, S. Y. Hoi, Pressure sensing and pressure redistribution mattress for prevention and management of pressure injuries. BMJ Innov. 9, 124–127 (2023).

[13] A. Misaki, K. Imanishi, S.-I. Takasugi, M. Wada, S. Fukagawa, M. Furue, Body pressure sensing mattress for bedsore prevention. SEI Tech. Rev. 78, 95–99 (2014).

[14] W. Xu, M. Huang, N. Amini, J. Liu, L. He, M. Sarrafzadeh, Smart insole: A wearable system for gait analysis, in *Proceedings of the 5th international conference on pervasive technologies related to assistive environments* (2012), pp. 1–4.

[15] J. Meyer, B. Arnrich, J. Schumm, G. Troster, Design and modeling of a textile pressure sensor for sitting posture classification. IEEE Sens. J. 10, 1391–1398 (2010).

[16] W.-Y. Chang, C.-C. Chen, C.-C. Chang, C.-L. Yang, An enhanced sensing application based on a flexible projected capacitive-sensing mattress. Sensors 14, 6922–6937 (2014). 10.3390/s140406922 PMC4029628 24747734

[17] A. Aghaei, S. Sajjadieh, B. Ghalebi, F. Irannejad, M. Shanbeh, A single-electrode, textile-based, flexible capacitive pressure sensor array. Eng. Proc. 52, 13 (2024).

[18] E. Marenzi, R. Lombardi, G. M. Bertolotti, A. Cristiani, B. Cabras, Design and development of a novel capacitive sensor matrix for measuring pressure distribution, in *2012 IEEE Sensors Applications Symposium Proceedings* (IEEE, 2012), pp. 1–6.

[19] J. Cheng, M. Sundholm, B. Zhou, M. Hirsch, P. Lukowicz, Smart-surface: Large scale textile pressure sensors arrays for activity recognition. Pervasive Mob. Comput. 30, 97–112 (2016).

[20] L. Dhakar, S. Gudla, X. Shan, Z. Wang, F. E. H. Tay, C. H. Heng, C. Lee, Large scale triboelectric nanogenerator and self-powered pressure sensor array using low cost roll-to-roll UV embossing. Sci. Rep. 6, 22253 (2016). 10.1038/srep22253 PMC4764913 26905285

[21] Y. Duan, S. He, J. Wu, B. Su, Y. Wang, Recent progress in flexible pressure sensor arrays. Nanomaterials 12, 2495 (2022). 10.3390/nano12142495 PMC9319019 35889718

[22] J.-S. Yang, M.-K. Chung, J.-Y. Yoo, M.-U. Kim, B.-J. Kim, M.-S. Jo, S.-H. Kim, J.-B. Yoon, Interference-free nanogap pressure sensor array with high spatial resolution for wireless human-machine interfaces applications. Nat. Commun. 16, 2024 (2025). 10.1038/s41467-025-57232-8 PMC11868543 40016221

[23] D. S. Thomas, S. W. Gilbert, Costs and cost effectiveness of additive manufacturing. NIST Special Publication 1176, 12 (2014).

[24] M. S. Sarwar, Y. Dobashi, C. Preston, J. K. M. Wyss, S. Mirabbasi, J. D. W. Madden, Bend, stretch, and touch: Locating a finger on an actively deformed transparent sensor array. Sci. Adv. 3, e1602200 (2017). 10.1126/sciadv.1602200 PMC5351976 28345045

[25] J. W. Kwak, M. Han, Z. Xie, H. U. Chung, J. Y. Lee, R. Avila, J. Yohay, X. Chen, C. Liang, M. Patel, I. Jung, J. Kim, M. Namkoong, K. Kwon, X. Guo, C. Ogle, D. Grande, D. Ryu, D. H. Kim, S. Madhvapathy, C. Liu, D. S. Yang, Y. Park, R. Caldwell, A. Banks, S. Xu, Y. Huang, S. Fatone, J. A. Rogers, Wireless sensors for continuous, multimodal measurements at the skin interface with lower limb prostheses. Sci. Transl. Med. 12, eabc4327 (2020). 10.1126/scitranslmed.abc4327 PMC7775724 33328330

[26] Y. Park, K. Kwon, S. S. Kwak, D. S. Yang, J. W. Kwak, H. Luan, T. S. Chung, K. S. Chun, J. U. Kim, H. Jang, H. Ryu, H. Jeong, S. M. Won, Y. J. Kang, M. Zhang, D. Pontes, B. R. Kampmeier, S. H. Seo, J. Zhao, I. Jung, Y. Huang, S. Xu, J. A. Rogers, Wireless, skin-interfaced sensors for compression therapy. Sci. Adv. 6, eabe1655 (2020). 10.1126/sciadv.abe1655 PMC7821894 33277263

[27] C. K. Franz, N. K. Murthy, G. R. Malik, J. W. Kwak, D. D’Andrea, A. R. Wolfe, E. Farr, M. A. Stearns, S. Deshmukh, J. O. Tavee, F. Sun, K. N. Swong, L. Rydberg, R. J. Cotton, L. F. Wolfe, J. M. Walter, J. M. Coleman III, J. A. Rogers, The distribution of acquired peripheral nerve injuries associated with severe COVID-19 implicate a mechanism of entrapment neuropathy: A multicenter case series and clinical feasibility study of a wearable, wireless pressure sensor. J. Neuroeng. Rehabil. 19, 108 (2022). 10.1186/s12984-022-01089-1 PMC9547638 36209094

[28] S. Celozzi, R. Araneo, P. Burghignoli, G. Lovat, Electromagnetic Shielding: Theory and Applications (John Wiley & Sons, 2022).

[29] A. A. Ivanov, A. V. Demakov, M. E. Komnatnov, T. R. Gazizov, On symmetry and asymmetry in nested electromagnetic shields. Symmetry 15, 441 (2023).

[30] X. C. Tong, Advanced Materials and Design for Electromagnetic Interference Shielding (CRC press, 2016).

[31] L. Hardell, M. Nilsson, T. Koppel, M. Carlberg, Aspects on the international commission on non-ionizing radiation protection (ICNIRP) 2020 guidelines on radiofrequency radiation. J. Cancer Sci. Clin. Ther. 05, 250–285 (2021).

[32] Y. H. Jung, J.-Y. Yoo, A. Vázquez-Guardado, J.-H. Kim, J.-T. Kim, H. Luan, M. Park, J. Lim, H.-S. Shin, C.-J. Su, R. Schloen, J. Trueb, R. Avila, J.-K. Chang, D. S. Yang, Y. Park, H. Ryu, H.-J. Yoon, G. Lee, H. Jeong, J. U. Kim, A. Akhtar, J. Cornman, T.-I. Kim, Y. Huang, J. A. Rogers, A wireless haptic interface for programmable patterns of touch across large areas of the skin. Nat. Electron. 5, 374–385 (2022).

[33] M. E. Posthauer, M. Banks, B. Dorner, J. M. G. A. Schols, The role of nutrition for pressure ulcer management: National pressure ulcer advisory panel, European pressure ulcer advisory panel, and pan pacific pressure injury alliance white paper. Adv. Skin Wound Care 28, 175–188 (2015). 10.1097/01.ASW.0000461911.31139.62 25775201

[34] A. S. Gorgey, D. R. Gater Jr., Prevalence of obesity after spinal cord injury. Top. Spinal Cord Inj. Rehabil. 12, 1–7 (2007). 10.1310/sci1204-1 PMC5818981 29472754

[35] A. Kovindha, P. Kammuang-Lue, P. Prakongsai, T. Wongphan, Prevalence of pressure ulcers in Thai wheelchair users with chronic spinal cord injuries. Spinal Cord 53, 767–771 (2015). 10.1038/sc.2015.77 PMC5399151 25939607

[36] Z. Li, F. Lin, L. Thalib, W. Chaboyer, Global prevalence and incidence of pressure injuries in hospitalised adult patients: A systematic review and meta-analysis. Int. J. Nurs. Stud. 105, 103546 (2020). 10.1016/j.ijnurstu.2020.103546 32113142

[37] S. Smith, A. Snyder, L. F. McMahon Jr., L. Petersen, J. Meddings, Success in hospital-acquired pressure ulcer prevention: A tale in two data sets. Health Aff. 37, 1787–1796 (2018). 10.1377/hlthaff.2018.0712 PMC6645390 30395514

[38] K.-H. Lee, Y. E. Kwon, H. Lee, Y. Lee, J. Seo, O. Kwon, S. W. Kang, D. Lee, Active body pressure relief system with time-of-flight optical pressure sensors for pressure ulcer prevention. Sensors 19, 3862 (2019). 10.3390/s19183862 PMC6766802 31500195

[39] S. Arias, E. Cardiel, L. Garay, B. Tovar, M. Pla, P. Rogeli, A pressure distribution measurement system for supporting areas of wheelchair users, in *2013 35th Annual International Conference of the IEEE Engineering in Medicine and Biology Society (EMBC)* (IEEE, 2013), pp. 4751–4754. 10.1109/EMBC.2013.6610609 24110796

[40] V. K. Nandikolla, R. Bochen, S. Meza, A. Garcia, Experimental gait analysis to study stress distribution of the human foot. J. Med. Eng. 2017, 3432074 (2017). 10.1155/2017/3432074 PMC5694576 29230404

[41] Z. Zhang, X. Gui, Q. Hu, L. Yang, R. Yang, B. Huang, B. R. Yang, Z. Tang, Highly sensitive capacitive pressure sensor based on a micropyramid array for health and motion monitoring. Adv. Electron. Mater. 7, 2100174 (2021).

[42] S.-J. Woo, J.-H. Kong, D.-G. Kim, J.-M. Kim, A thin all-elastomeric capacitive pressure sensor array based on micro-contact printed elastic conductors. J. Mater. Chem. C Mater. 2, 4415–4422 (2014).

[43] Y. Jeong, J. Gu, J. Byun, J. Ahn, J. Byun, K. Kim, J. Park, J. Ko, J. H. Jeong, M. Amjadi, I. Park, Ultra-wide range pressure sensor based on a microstructured conductive nanocomposite for wearable workout monitoring. Adv. Healthc. Mater. 10, e2001461 (2021). 10.1002/adhm.202001461 33694309

