# Supplementary material for: Monolithic and scalable capacitive electrodes leveraging parasitic capacitance for full-body pressure mapping
Source: Sci Adv. 2026 Oct 30;12(44):eaeh8618. doi: 10.1126/sciadv.aeh8618 (PMC13721990; doi:10.1126/sciadv.aeh8618)
Supplement: Supplementary file 1 [file sciadv.aeh8618_sm.pdf]

Science Advances
AAAS

Supplementary Materials for

Monolithic and scalable capacitive electrodes leveraging parasitic capacitance for full-body pressure mapping

Donho Lee *et al.*

Corresponding author: Jungrak Choi, cjr1992@etri.re.kr; Inkyu Park, Inkyu@kaist.ac.kr

Sci. Adv. **12**, eaeh8618 (2026)
DOI: 10.1126/sciadv.aeh8618

The PDF file includes:

Figs. S1 to S20
Tables S1 to S7
Legends for movies S1 to S5

Other Supplementary Material for this manuscript includes the following:

Movies S1 to S5

Science Advances
AAAS

Supplementary Materials for

Monolithic and Scalable Capacitive Electrodes Leveraging Parasitic Capacitance for Full-Body Pressure Mapping

Donho Lee *et al.*

*Corresponding authors. E-mail: cjr1992@etri.re.kr (J. Choi); Inkyu@kaist.ac.kr (I. Park)

This PDF file includes:

Figs. S1 to S20
Tables S1 to S7
Movies S1 to S5

Other Supplementary Materials for this manuscript include the following:

Movies S1 to S5

Fig. S1.

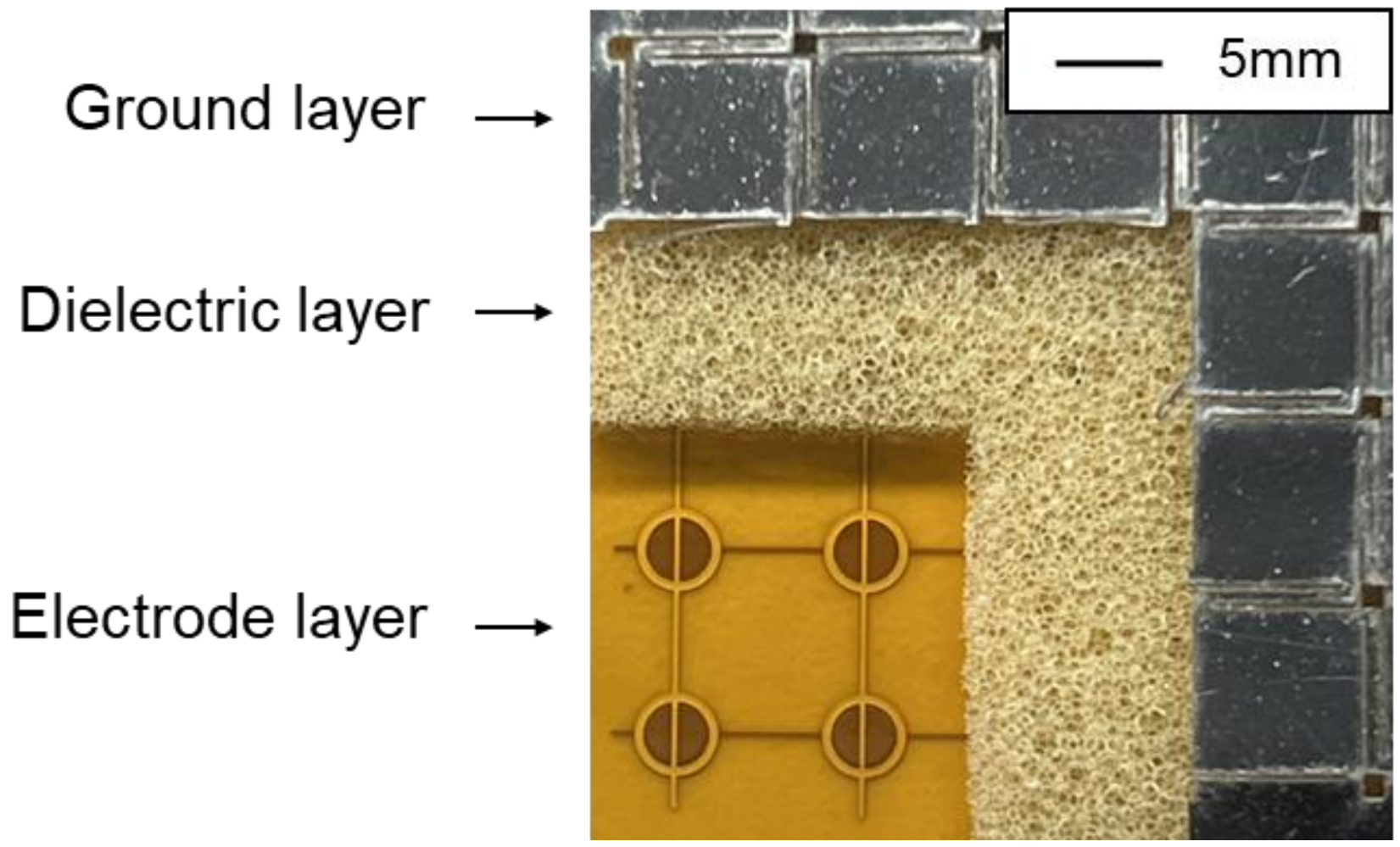

Photograph of pressure sensor array. A cut sensor consisting of a electrode layer (fPCB), a dielectric layer, and a ground (GND) layer.

Fig. S2.

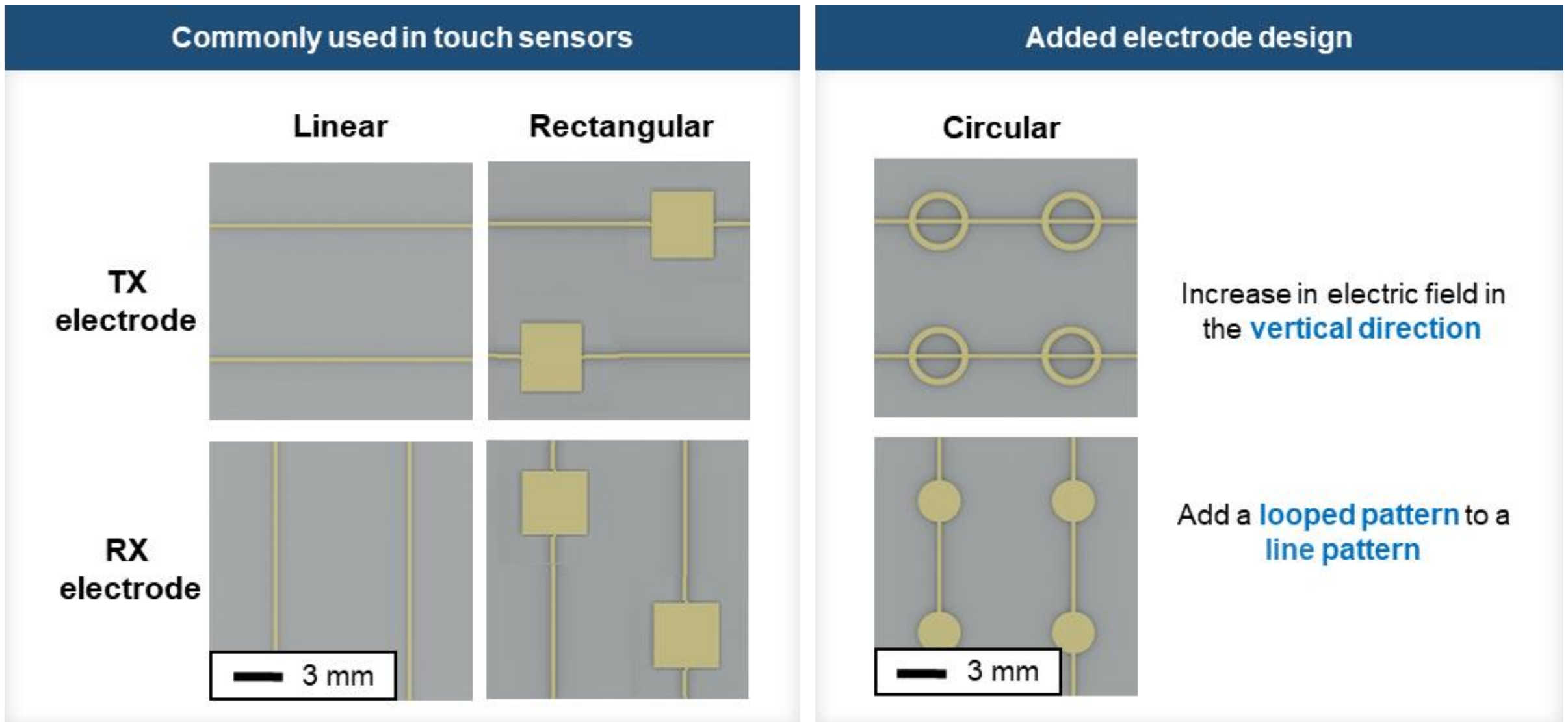

Monolithic TX-RX electrode layer. Three different TX-RX electrode designs: linear, rectangular, and circular patterns. The linear and rectangular patterns are commonly used in commercial touch sensors. The circular pattern designed to enhance the magnitude of the electric field.

Fig. S3.

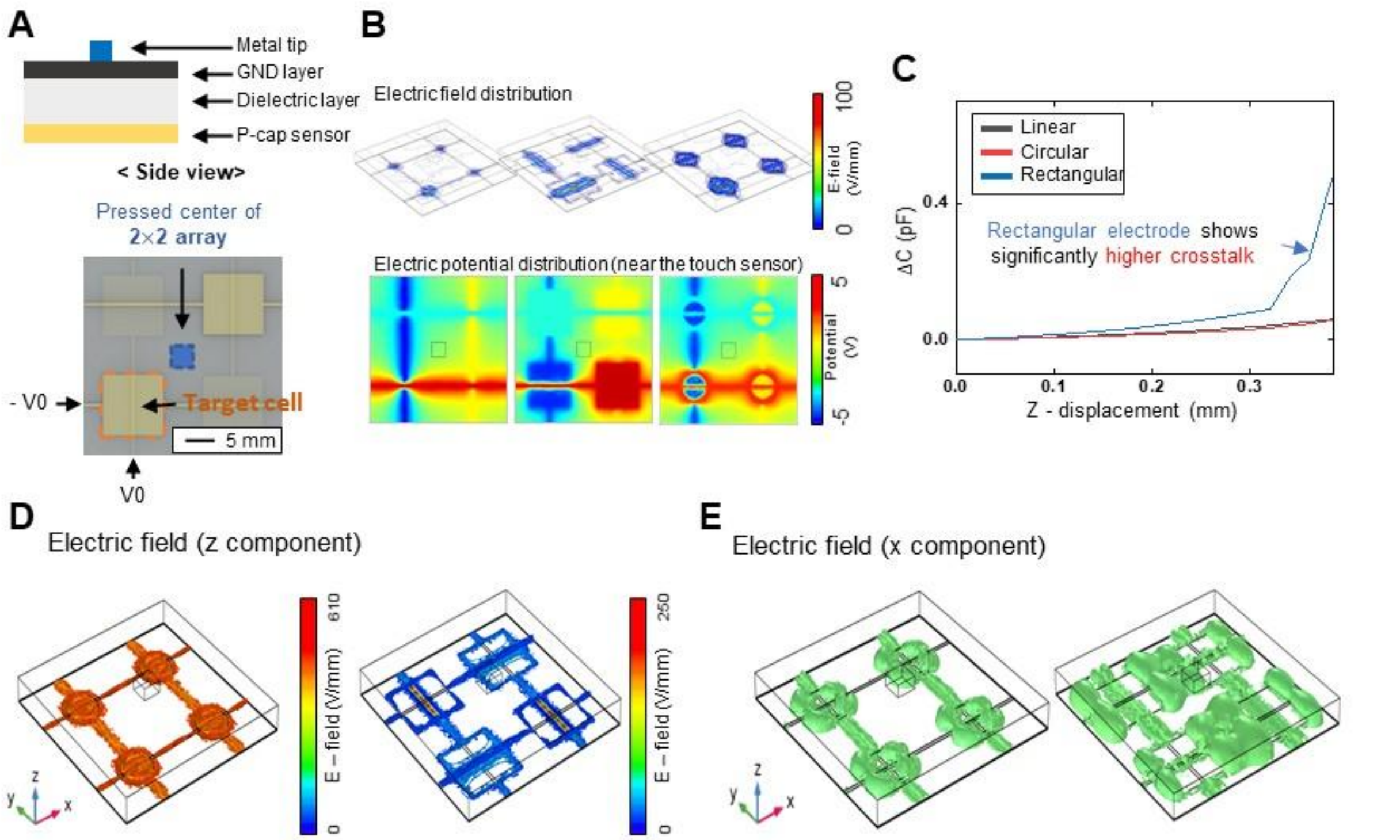

FEA Simulation results of crosstalk for the electrode layer design. (**A**) Schematic illustration of experimental setup for a 2×2 sensor array with the tip located at the center. (**B**) Electric field distribution and voltage distribution contours on the TX-RX electrode layer. (**C**) The change in capacitance of the rectangular electrode pattern is eight times larger than that of the other electrode patterns. (**D**) The circular pattern model, generating a strong vertical electric field, exhibits higher sensitivity compared to the rectangular pattern model. (**E**) The rectangular model, generating a strong horizontal electric field, exhibits larger crosstalk compared to the circular pattern model.

Fig. S4.

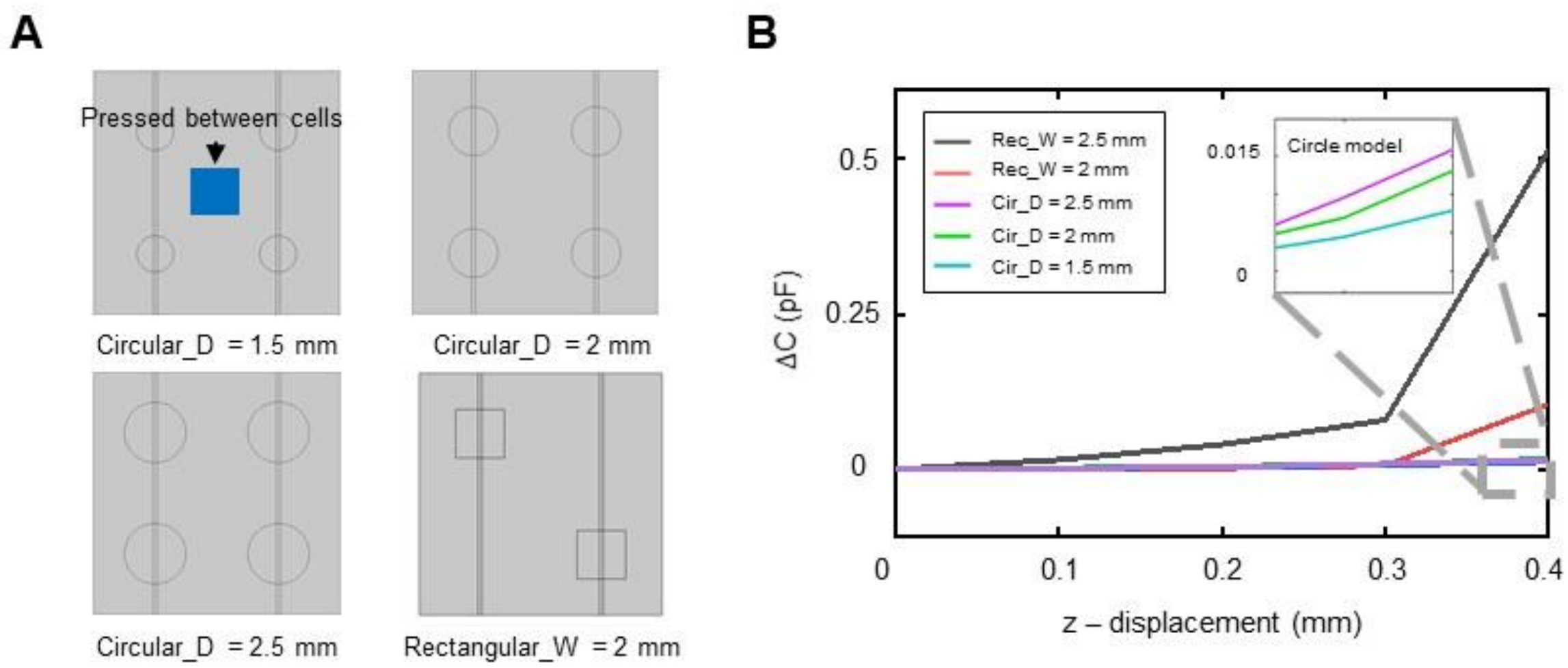

FEA Simulation results of crosstalk for electrode models of different sizes. (**A**) Electrode pattern designs, including circular pattern designs (D = 1.5, 2.0, and 2.5 mm) and a rectangular pattern design (W = 2.0 mm), were used for comparison. (**B**) Simulation results indicate that rectangular models exhibit higher crosstalk, while the circular model remains stable.

Fig. S5.

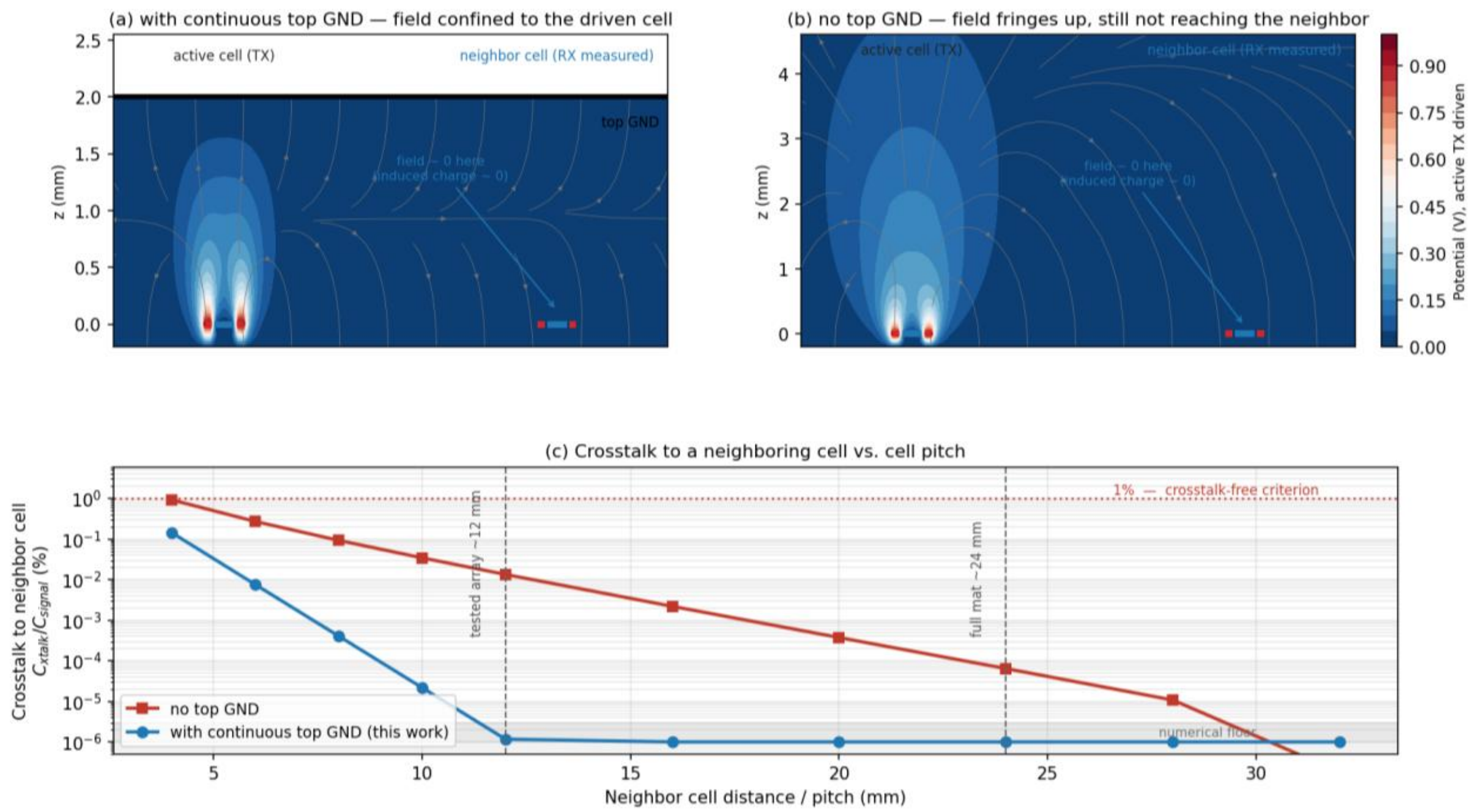

Simulated electrical crosstalk between neighboring cells versus cell spacing, with and without the grounded top plane. Representative benchtop spacing; at the larger deployed array spacing the coupling is even smaller**.**

Fig. S6.

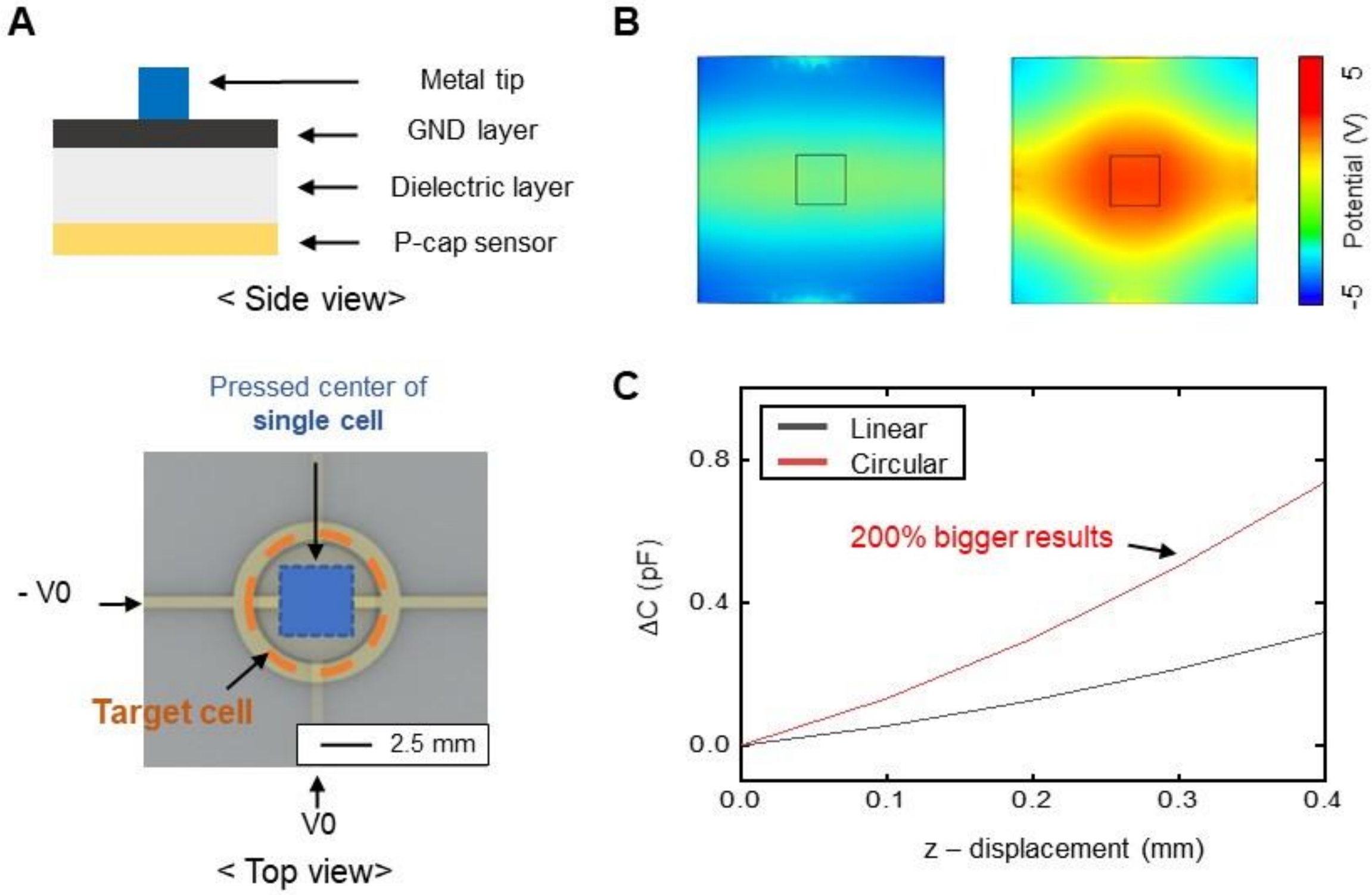

FEA Simulation results comparing the sensitivity of electrode pattern designs. (**A**) Schematic illustration of modeling with a single sensor and tip that moves vertically to the cell center. (**B**) Voltage distribution of the circular pattern model (right), which is larger than that of the linear pattern model (left). (**C**) The change in capacitance shows that the circular pattern model is twice as sensitive as the linear model.

Fig. S7.

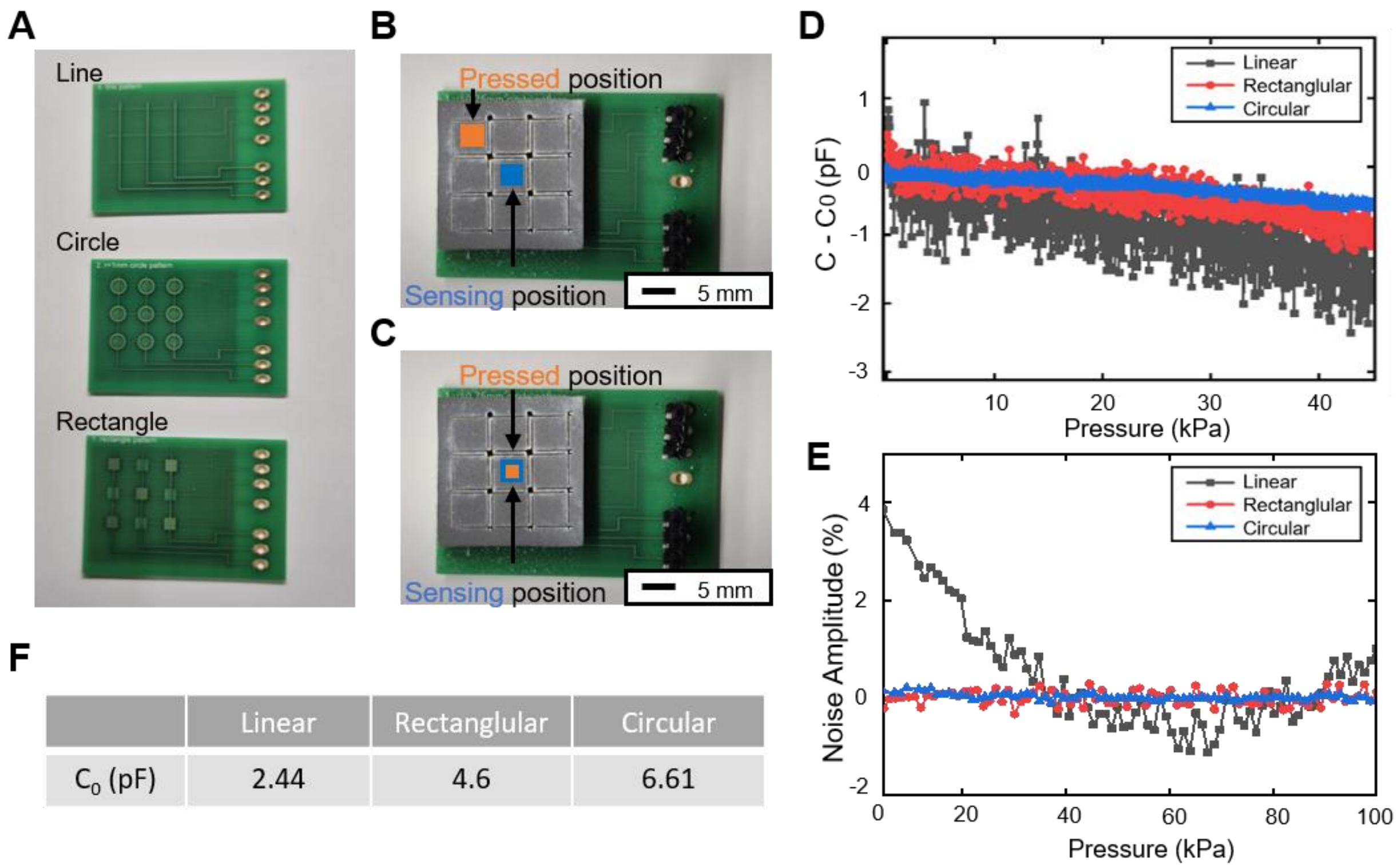

	Linear	Rectanglular	Circular
C_0 (pF)	2.44	4.6	6.61

Experimental comparison of crosstalk and signal-to-noise (SNR) for different electrode layer models. (**A**) Photographs of the three sensor array designs: linear, circular (D = 2 mm), and rectangular (W = 2 mm) patterns. (b, c) The measurement setup showing the pressing spot and sensing position. (**D**) Experimental results of the crosstalk test. The linear pattern shows the largest and unstable capacitance change. (**E**) Noise amplitude comparison. The circular pattern demonstrates superior stability with negligible noise, whereas the linear pattern exhibits significant fluctuations. (**F**) Baseline capacitance (C_0) values measured for each electrode model.

Fig. S8.

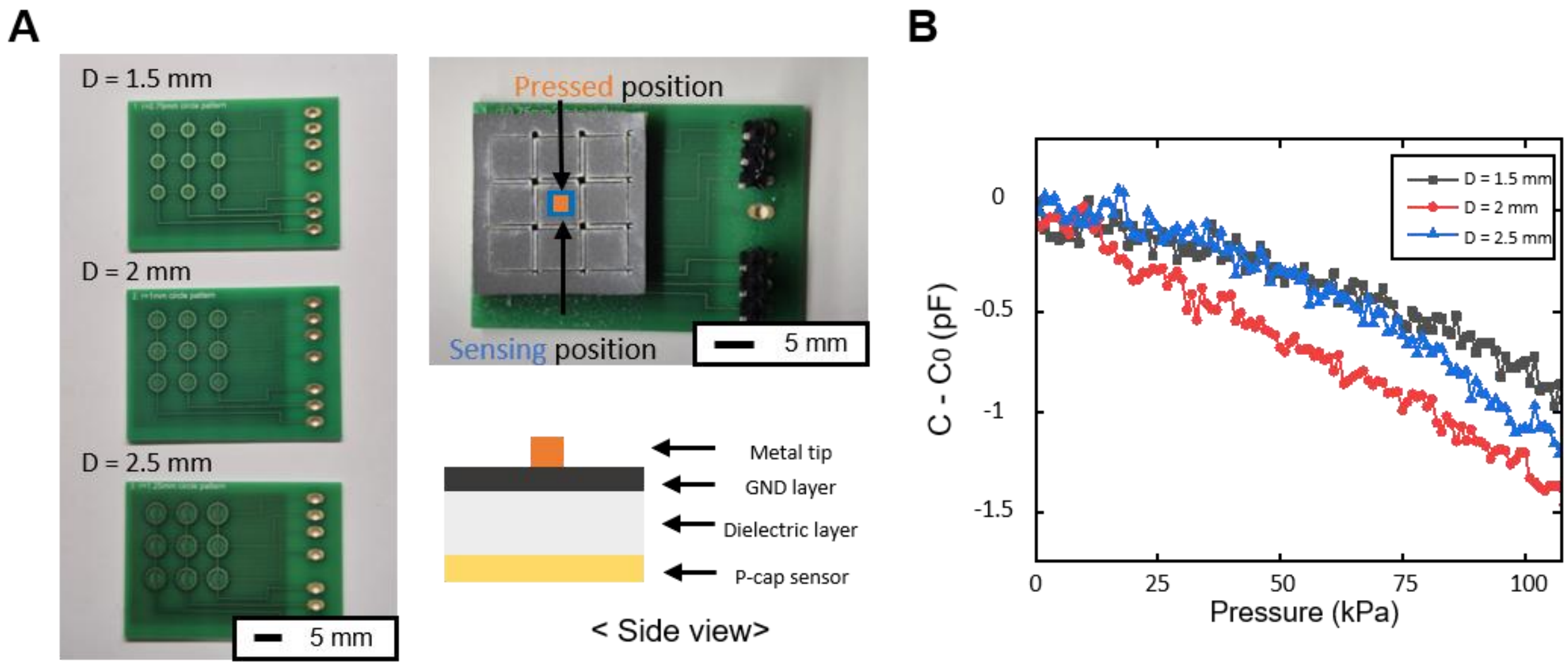

Experimental comparison of the sensitivity of the circular electrode models with different diameters. (**A**) Photographs of the three sensor array designs with circular patterns (D = 1.5 mm, 2 mm, and 2.5 mm). (**B**) Experimental results on sensitivity. The pattern with D = 2 mm shows the largest capacitance change.

Fig. S9.

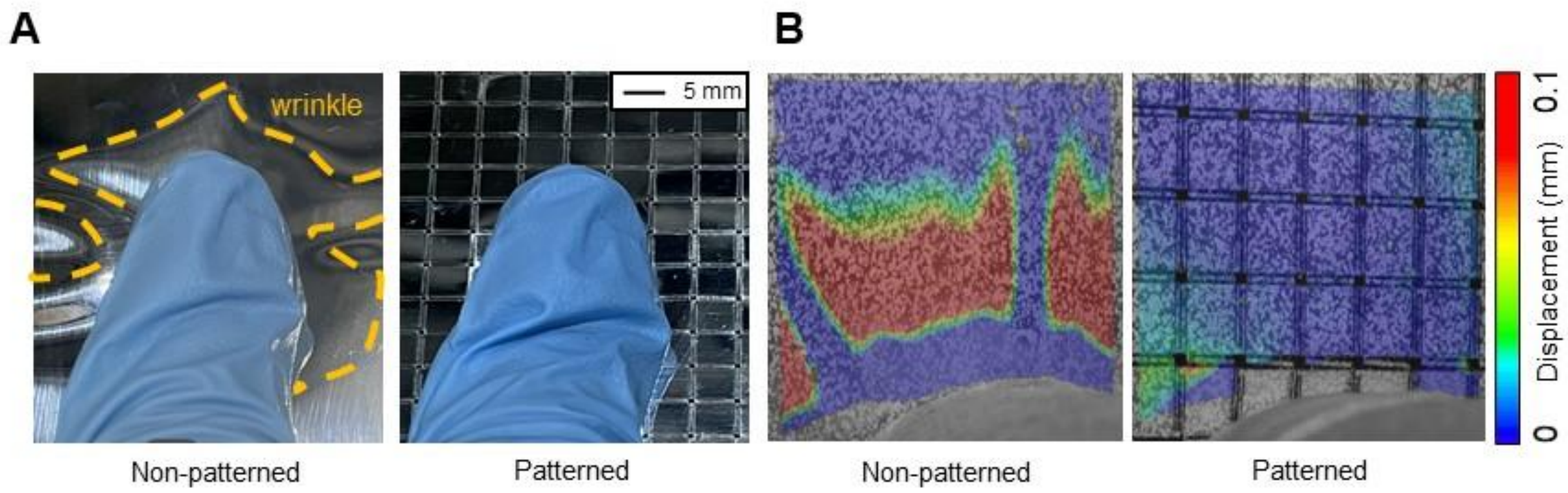

Comparison of the GND layer before and after laser cutting. (**A**) The uncut GND layer wrinkles under applied pressure due to lack of stretchability, while the laser-cut GND layer deforms without wrinkling. (**B**) DIC results for the GND layer with and without laser cutting process.

Fig. S10.

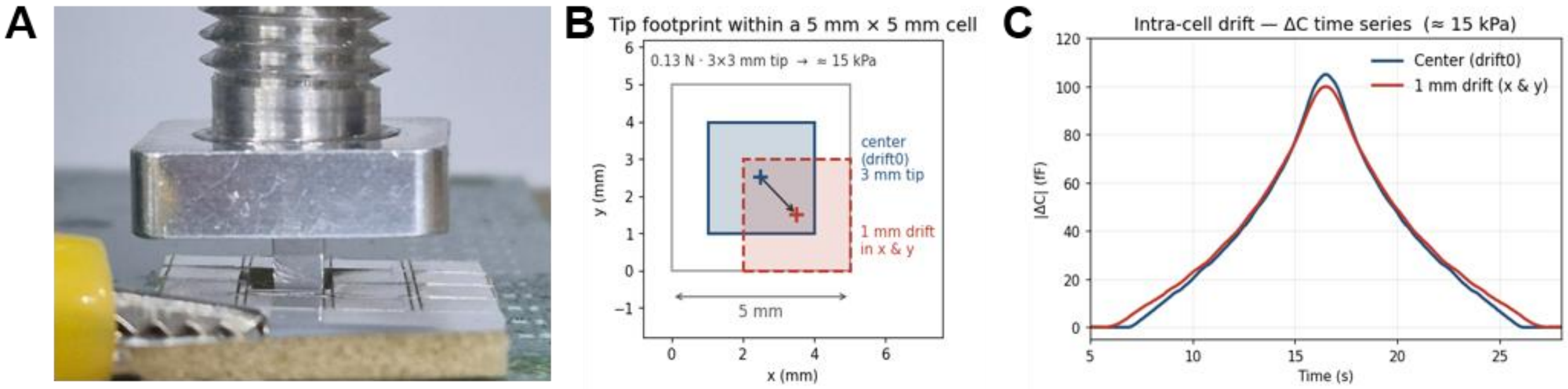

Sensor response when pressure was applied 1 mm diagonally offset from the center (edge condition). (**A**) Photograph of the pressure sensor under pressure at the edge. (**B**) Top-view of the location where the pressure tip was applied. (**C**) Comparison of the sensor response with respect to the location (i.e., center and edge).

Fig. S11.

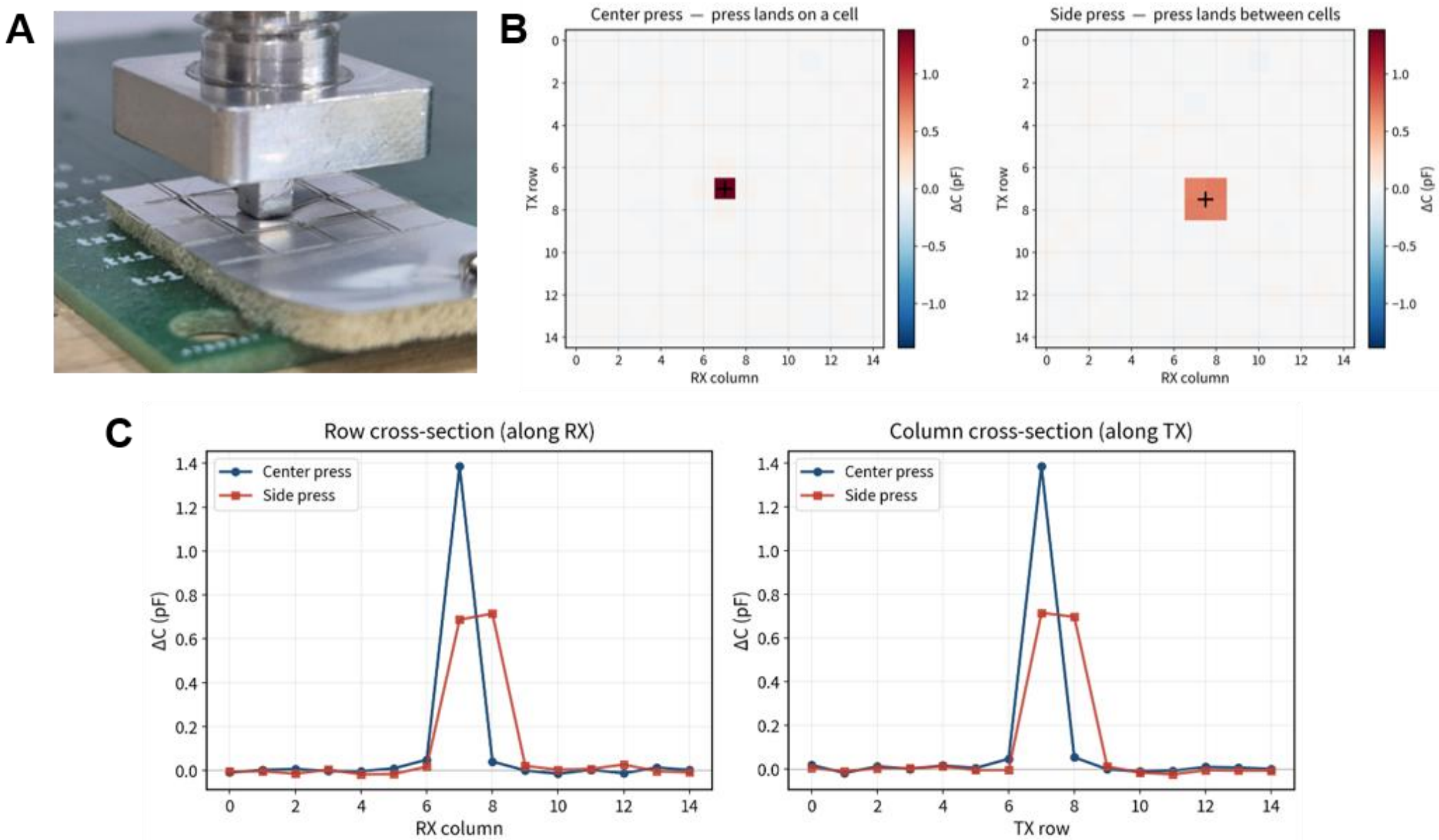

Sensor response when pressure was applied at the corner of a section. (**A**) Photograph of the pressure sensor under pressure at the corner. (**B**) Top-view schematic of the pressure tip locations at the center and corner. (**C**) Comparison of the sensor response (RX and TX electrodes) with respect to the location (i.e., center and corner).

Fig. S12.

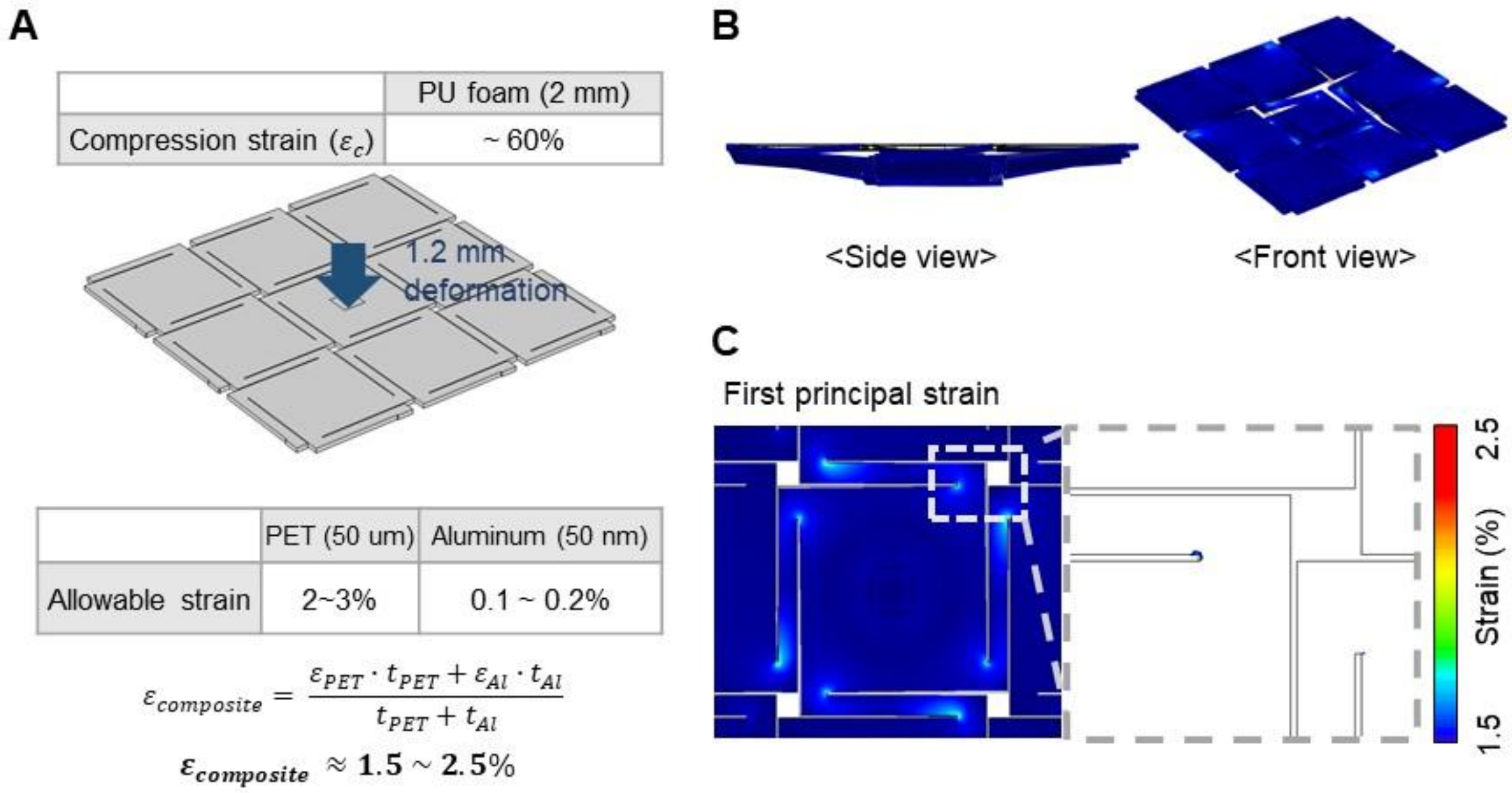

FEA simulation results for GND layer deformation. (**A**) Schematic illustration of PU foam under 60% compressive strain. (**B**) Strain distribution contour of Al-deposited PET showing elastic deformation. (**C**) Maximum strain occurs at the legs of the electrode pattern, within the elastic range.

Fig. S13.

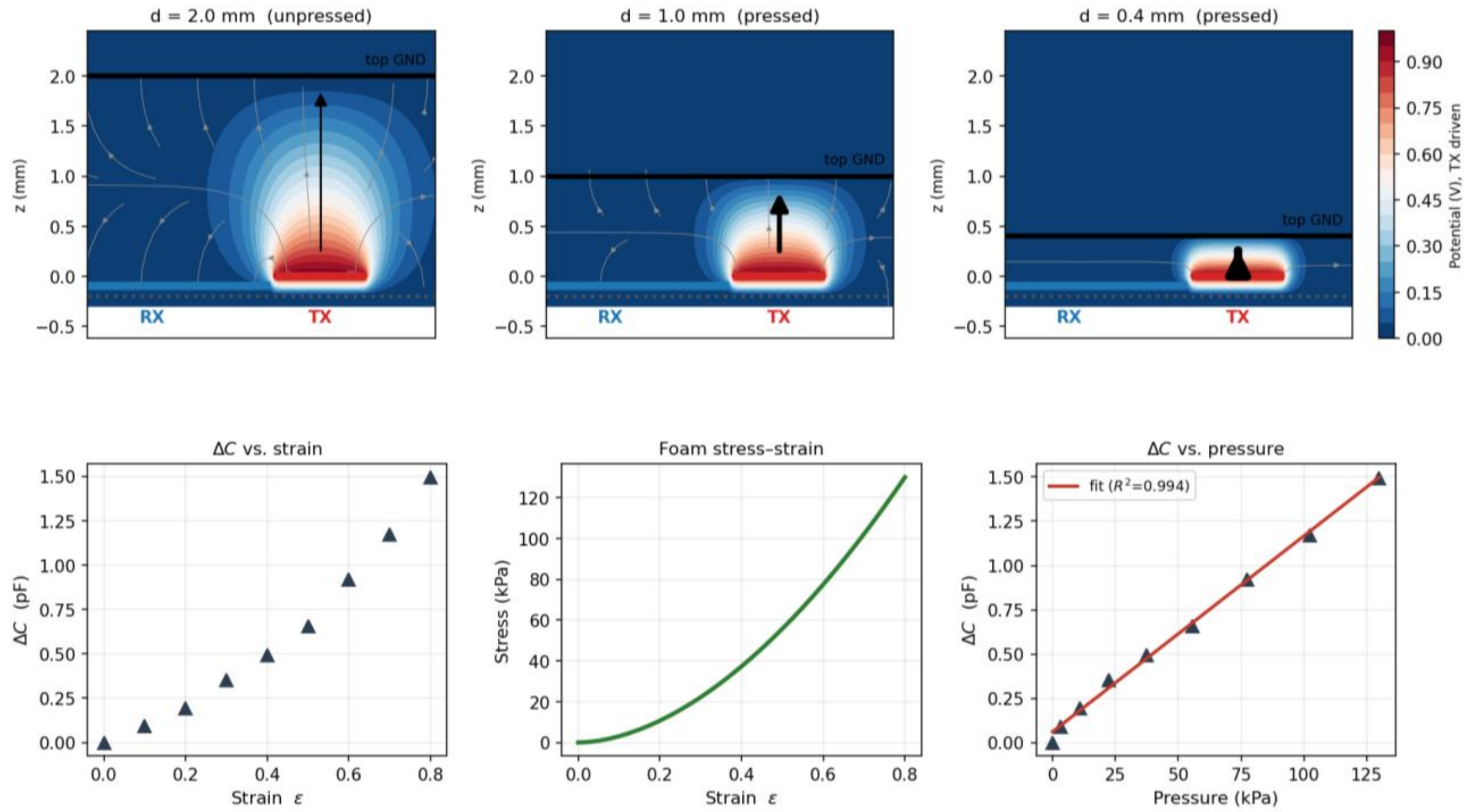

Electrostatic field simulation of the single cell as the top ground plane descends. Only the parasitic coupling to the top plane is modelled, and the resulting ΔC is linear in pressure (R^2 = 0.994).

Fig. S14.

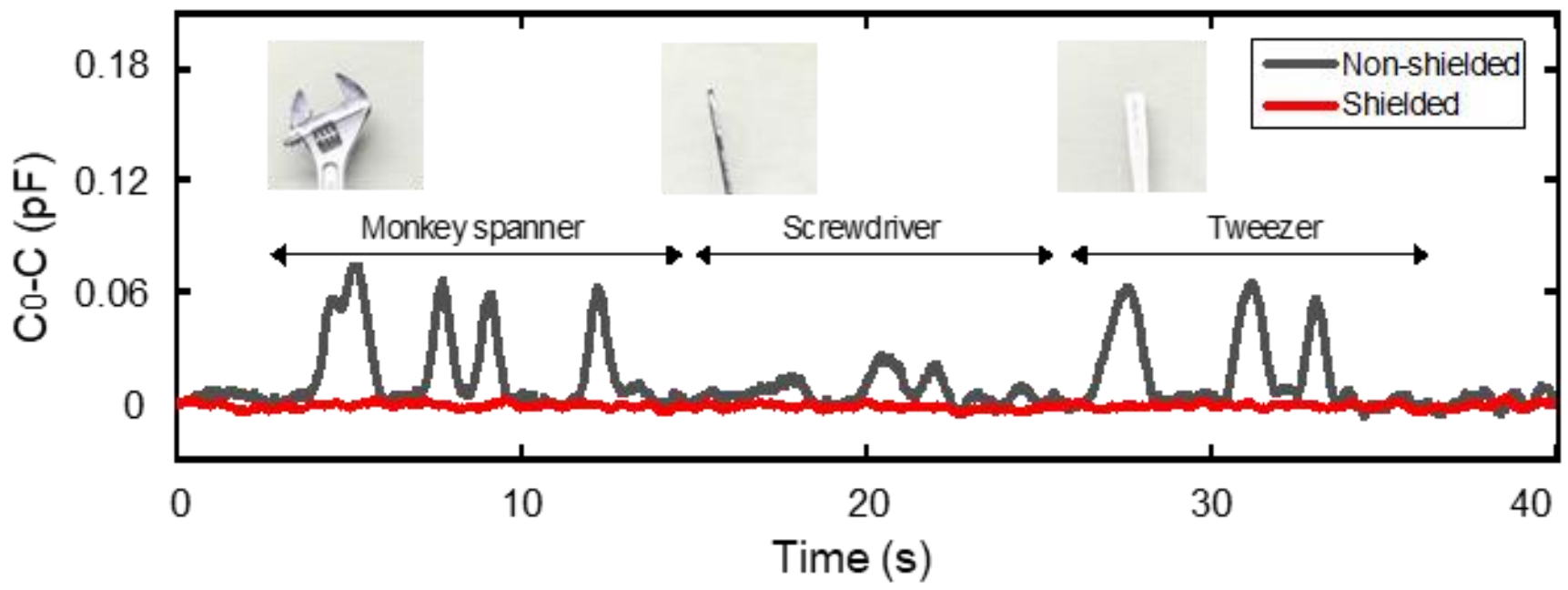

Performance characteristics of pressure sensor. Signal noise measured by hovering various conductive objects, such as monkey spanner, screwdriver, and tweezer, over the pressure sensor.

Fig. S15.

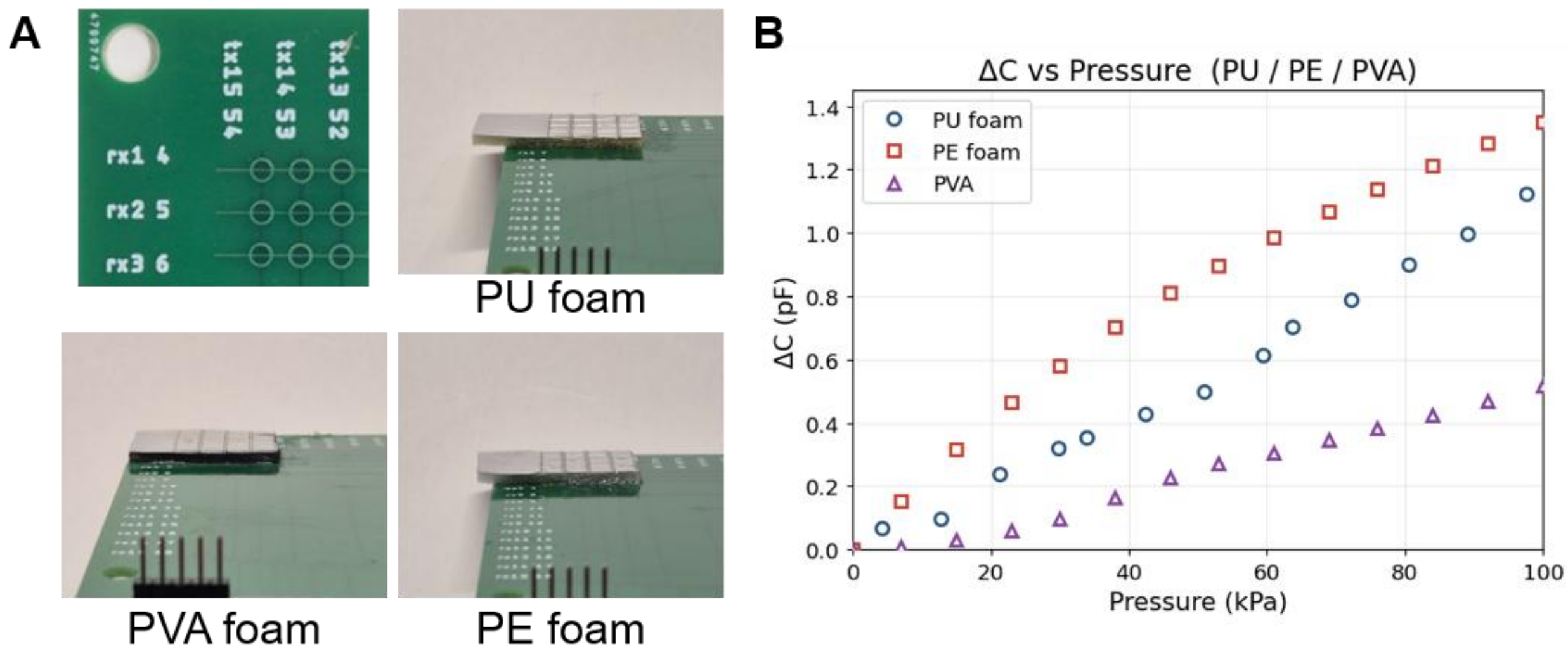

Characteristics of the pressure sensor with different dielectric materials. (**A**) 3 × 3 array TX-RX electrode, which was covered by polymeric dielectric materials, Polyurethane, Polyvinyl Alcohol, and Polyethylene. **(B)** Pressure sensor responses with different dielectric materials.

Fig. S16.

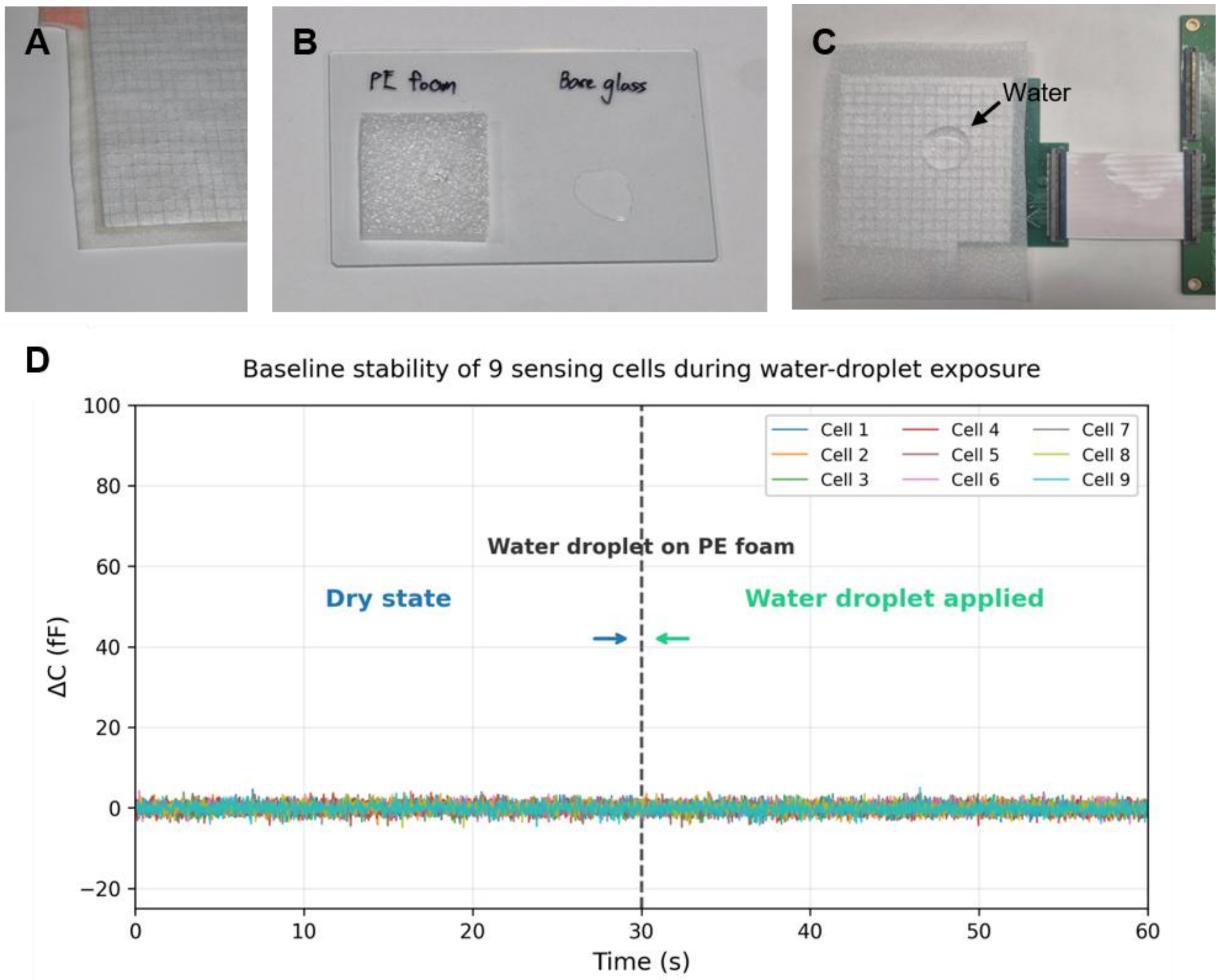

Water-insensitive property of the pressure sensor array using a hydrophobic PE (Polyethylene) foam outer layer. (**A**) Photograph of the pressure sensor array covered by the polyethylene (PE) foam protective outer layer. (**B**) Comparison of water droplet behavior on PE foam and bare glass surfaces, demonstrating the hydrophobicity of PE. (**C**) The sensor with the PE-foam outer layer and a water droplet placed on top of the PE foam. (**D**) Baseline capacitance of nine sensing cells read out at 30 Hz. No shift, drift, or transient response is observed during the experiment.

Fig. S17.

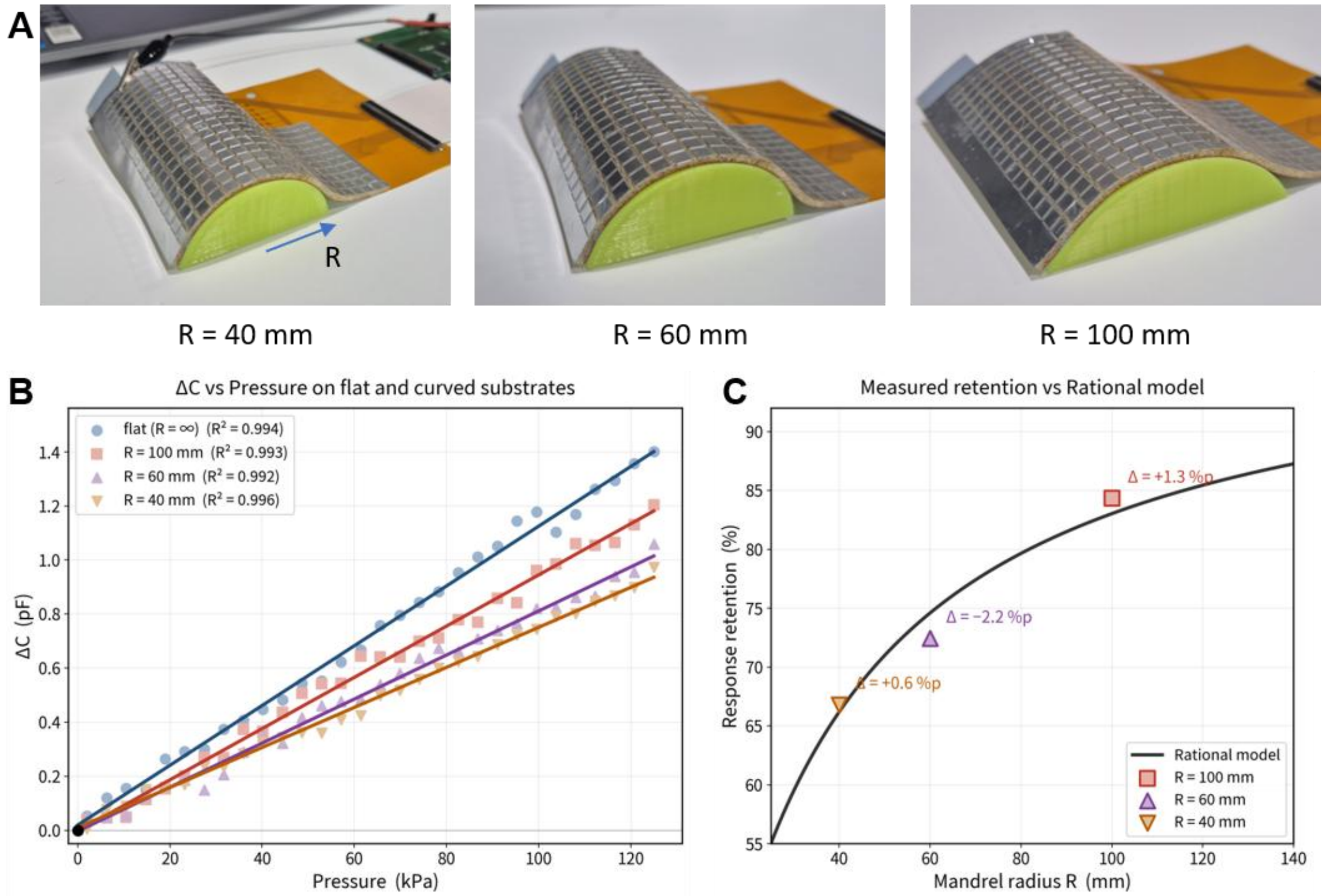

Pressure-sensing performance on flat and singly-curved (R = 40 / 60 / 100 mm) substrates. (**A**) Photographs of a sensor coupon conformed to the curved mandrels. (**B**) Pressed-cell ΔC versus applied pressure on each substrate; the response is linear on all four substrates ($R^2 \geq 0.99$), and the slope decreases with decreasing radius. (**C**) Response retention versus mandrel radius, well described by a one-parameter saturating calibration relation that enables per-radius re-baselining for singly-curved deployment.

Fig. S18.

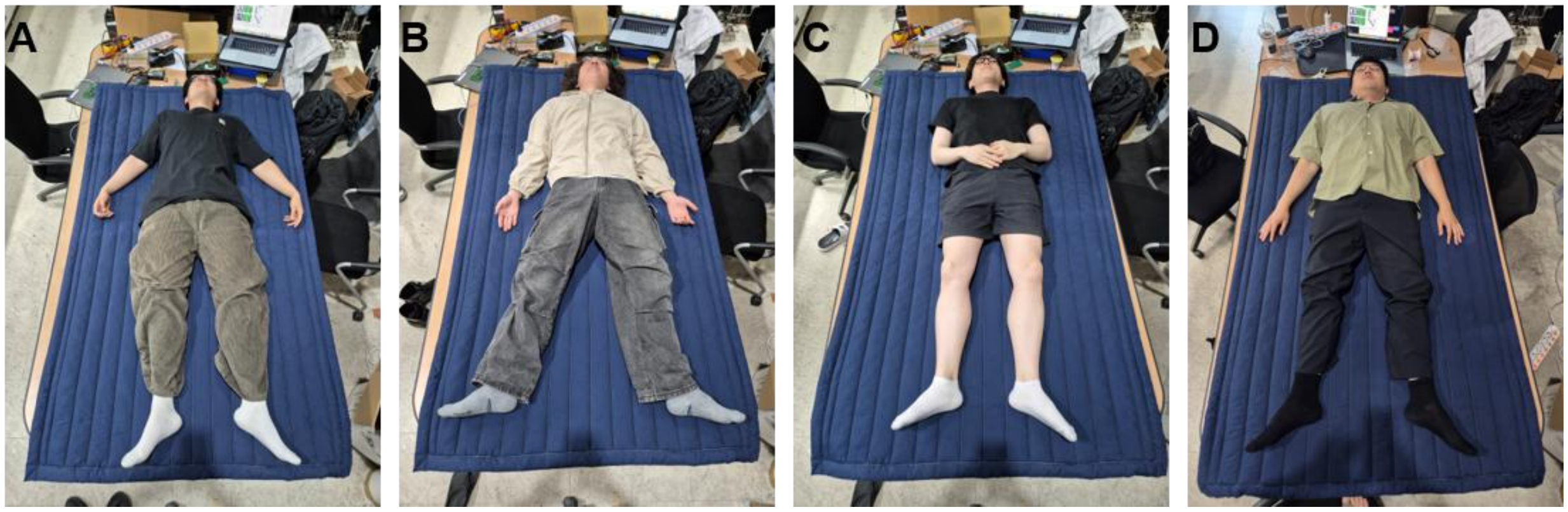

Photographs of the four additional subjects (S02–S05) lying on the sensing mattress for the posture-classification study. (**A**) 31-year-old male; height 176 cm; mass 83 kg; BMI 26.8. (**B**) 28-year-old male; height 176 cm; mass 80 kg; BMI 25.8. (**C**) 26-year-old male; height 174 cm; mass 75 kg; BMI 24.8. (**D**) 31-year-old male; height 174 cm; mass 80 kg; BMI 26.4.

Fig. S19.

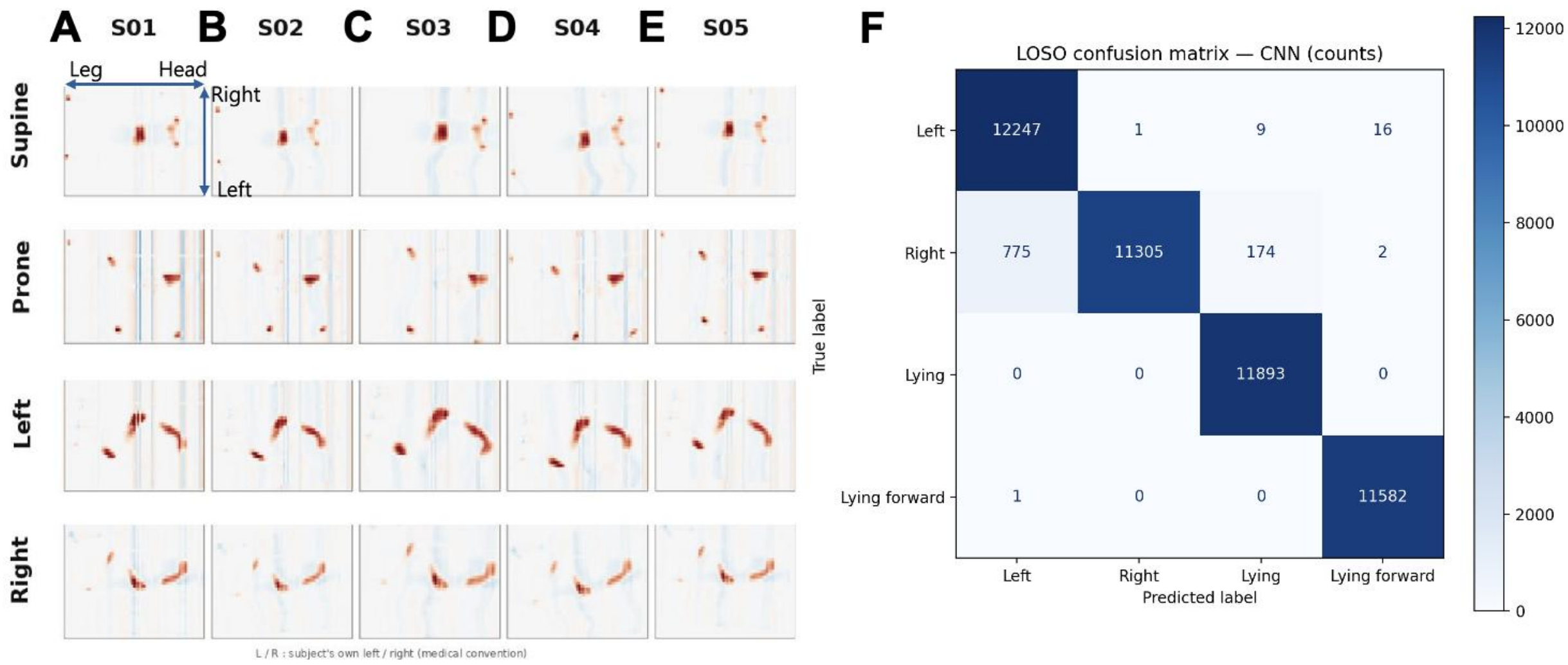

Subject-independent posture classification. (**A**)–(**E**) Representative pressure-map snapshots for each of the four target postures (rows: Lying, Lying forward, Left, Right) for the five subjects (columns: a = S01, b = S02, c = S03, d = S04, e = S05). (**F**) Confusion matrix (counts) aggregated over all five LOSO folds (48,005 test predictions).

Fig. S20.

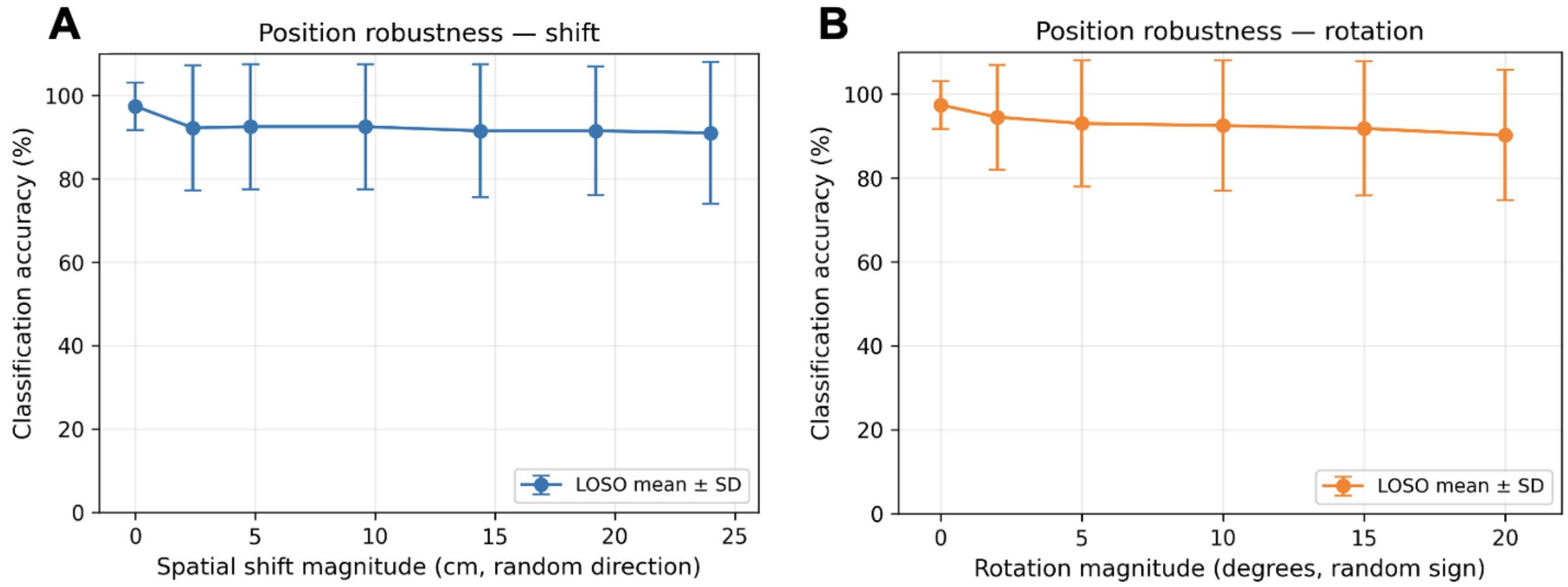

Deployment robustness of the LOSO-trained classifier under test-time spatial transforms (no retraining). (**A**) Classification accuracy versus spatial shift magnitude Δ; each test frame is translated by random row/column offsets uniformly drawn from −Δ to +Δ cm on the 1.7 m mat. (**B**) Classification accuracy versus rotation magnitude θ; each test frame is rotated by an angle uniformly drawn from −θ to +θ°. Markers and error bars are LOSO mean ± SD across the five folds; the larger SD is dominated by the S03 fold (per-fold baseline accuracies: S01, 99.95%; S02, 100.00%; S03, 87.27%; S04, 100.00%; S05, 99.83%). A joint (shift × rotation) sweep across the four folds excluding S03 is provided in Table S7.

Table S1.

Comparison of GND layer materials. Al-PET with aluminum deposition offers low cost, stable material properties, and manufacturing process advantages for mass production.

	Al-PET	ITO film	Ag nanowire film
Conductivity (S/m)	2.82×10^7	1×10^5	6.3×10^7
Cost ($\$/m^2$)	1 ~ 3	20 ~ 100	10 ~ 70
Flexibility	Flexible	-	Flexible
Durability	Excellent	Sensitive to mechanical damage	Oxidation of surfaces
Ease of production	Mass-producible	Limited	Experimental stage

Table S2.

Table of dielectric layer materials. Comparison of the dielectric materials used in capacitive pressure sensors.

	PU foam (2 mm)	PDMS	EVA foam	PVA foam	PE foam
Dielectric constant (ε_r)	1.1–1.5	2.6–2.8	1.8–2.3	3.0–5.0	1.1–1.5
Compression strain (ε_c)	~ 60%	~ 20%	~ 50%	~35%	~55%
Compression modulus (E_c)	0.03 - 0.1 MPa	0.3 - 1.5 MPa	0.05 - 0.2 MPa	0.1 - 0.5 MPa	0.05 - 0.2 MPa
Cost	5 $	200 - 300 $	10 - 20 $	10 - 20 $	10 - 20 $

Table S3.

Operating frequencies and wavelengths of common electronic devices in daily life. This table summarizes frequency ranges and wavelengths (λ) of electronic devices. The maximum slit width (approximately 1.2 mm) induced by mechanical deformation of the patterned GND layers remain significantly below the recommended slit limit (λ/50).

Device / Technology	Operating Frequency	Wavelength (λ)
AM radio broadcasting	530–1700 kHz	~ 176–566 m
RFID (High Frequency)	13.56 MHz	~22.1 m
FM radio broadcasting	88–108 MHz	~2.8–3.4 m
RFID (UHF)	860–960 MHz	~0.31–0.35 m
Cellular (LTE)	700 MHz – 2.6 GHz	~0.11–0.43 m
GPS	1.575 GHz	~0.19 m
Bluetooth, Wi-Fi (802.11b/g/n)	2.4 GHz	~0.125 m
Wi-Fi (802.11a/ac), Radar, 5G	5 GHz	~0.06 m
Microwave oven	2.45 GHz	~0.122 m

Table S4.

International safety standards for electromagnetic fields based on operating frequency. Table shows the ICNIRP safety limits for electric and magnetic field strength in the 100 kHz–1 MHz frequency range. The touch sensor used in this study is based on mXT2912 controller, which typically operates around 100-500 kHz. To evaluate electromagnetic safety, the electric (E-field) and magnetic (B-field) emissions from the sensor were measured, resulting in 74 V/m and 0.12 µT, respectively. These values are significantly below the ICNIRP limits for 500 kHz (1,220 V/m and 320.0 µT), confirming compliance with international safety standards.

Frequency	E-field limit (V/m)	B-field limit (µT)
100 kHz (0.1 MHz)	6100.0 V/m	1600.0 µT
200 kHz (0.2 MHz)	3050.0 V/m	800.0 µT
300 kHz (0.3 MHz)	2033.3 V/m	533.3 µT
400 kHz (0.4 MHz)	1525.0 V/m	400.0 µT
500 kHz (0.5 MHz)	1220.0 V/m	320.0 µT
600 kHz (0.6 MHz)	1016.7 V/m	266.7 µT
700 kHz (0.7 MHz)	871.4 V/m	228.6 µT
800 kHz (0.8 MHz)	762.5 V/m	200 µT
900 kHz (0.9 MHz)	677.8 V/m	177.8 µT
1 MHz	610.0 V/m	160.0 µT

Table S5.
System-level comparison of the present architecture and iontronic capacitive sensing.

Criterion	Iontronic capacitive	This work
Per-cell signal magnitude	Large (nF–μF)	Small (pF)
Per-cell settling time	Long	Short
Switching power at array scale	High	Low
Excitation waveform	AC required (avoid electrolysis)	DC charge-transfer
AC drive current at array scale	Exceeds commercial IC ratings	Well within IC ratings
EMI rejection bandwidth	Low-frequency only (EDL-bounded)	Wide-band (continuous grounded top plane)
Commercial readout-IC ecosystem	Not available; custom ASIC required	Mature, mass-produced
Demonstrated scale	Lab-scale, small-area	m^2-scale, multi-thousand cells, video rate

Table S6.
Demographic and dataset composition of the five subjects (S01–S05) used in the LOSO cross-validation study.

Subject	Sex	Age (y)	Height (cm)	Weight (kg)	BMI	Test frames	Left	Right	Lying	Lying forward
**S01 (original)**	M	28	178	85	26.8	10,847	2,724	2,788	2,619	2,716
**S02**	M	31	176	83	26.8	9,962	2,546	2,573	2,497	2,346
**S03**	M	28	176	80	25.8	7,531	1,963	1,858	1,848	1,862
**S04**	M	26	174	75	24.8	11,289	2,961	2,857	2,869	2,602
**S05**	M	31	174	80	26.4	8,376	2,079	2,180	2,060	2,057
**Total**						**48,005**	**12,273**	**12,256**	**11,893**	**11,583**

Table S7.
Position-robustness sweep of the LOSO-trained classifier under joint (shift × rotation) test-time transforms without retraining. Values are mean accuracy across the four LOSO folds with baseline accuracy above 99% (held-out subjects S01, S02, S04, and S05); the S03 fold (baseline accuracy 87.27%) is not included. Results for all five folds, including S03, are shown in Fig. S20.

Spatial shift	0°	5°	15°	20°
0 cm	99.95%	99.89%	99.10%	97.33%
10 cm	99.13%	99.33%	97.62%	96.03%
19 cm	98.77%	98.96%	96.92%	95.41%
24 cm	98.58%	98.83%	96.71%	95.22%

Movie S1. **Shielding test result**

Movie S2. **Resolution test**

Movie S3. **Bedsore monitoring**

Movie S4. **Workout monitoring**

Movie S5. **Balance monitoring**
